# ﻿Maximising informativeness for target capture-based phylogenomics in *Erica* (Ericaceae)

**DOI:** 10.3897/phytokeys.251.136373

**Published:** 2025-01-16

**Authors:** Seth D. Musker, Nicolai M. Nürk, Michael D. Pirie

**Affiliations:** 1 Department of Biological Sciences, University of Cape Town, Rondebosch, Cape Town, South Africa University of Bayreuth Bayreuth Germany; 2 Department of Plant Systematics, Bayreuth Centre of Ecology and Environmental Research (BayCEER), University of Bayreuth, Universitätsstraße 30, 95447, Bayreuth, Germany University of Cape Town Cape Town South Africa; 3 University Museum, The University of Bergen, Postboks 7800, N-5020, Bergen, Norway The University of Bergen Bergen Norway

**Keywords:** Bioinformatics, Ericaceae, Phylogenomics, Target capture

## Abstract

Plant phylogenetics has been revolutionised in the genomic era, with target capture acting as the primary workhorse of most recent research in the new field of phylogenomics. Target capture (aka Hyb-Seq) allows researchers to sequence hundreds of genomic regions (loci) of their choosing, at relatively low cost per sample, from which to derive phylogenetically informative data. Although this highly flexible and widely applicable method has rightly earned its place as the field’s *de facto* standard, it does not come without its challenges. In particular, users have to specify which loci to sequence—a surprisingly difficult task, especially when working with non-model groups, as it requires pre-existing genomic resources in the form of assembled genomes and/or transcriptomes. In the absence of taxon-specific genomic resources, target sets exist that are designed to work across broad taxonomic scales. However, the highly conserved loci that they target may lack informativeness for difficult phylogenetic problems, such as that presented by the rapid radiation of *Erica* in southern Africa. We designed a target set for *Erica* phylogenomics intended to maximise informativeness and minimise paralogy while maintaining universality by including genes from the widely used Angiosperms353 set. Comprising just over 300 genes, the targets had excellent recovery rates in roughly 90 *Erica* species as well as outgroups from *Calluna*, *Daboecia*, and *Rhododendron*, and had high information content as measured by parsimony informative sites and Quartet Internode Resolution Probability (QIRP) at shallow nodes. Notably, QIRP was positively correlated with intron content, while including introns in targets—rather than recovering them via exon-flanking “bycatch”—substantially improved intron recovery. Overall, our results show the value of building a custom target set, and we provide a suite of open-source tools that can be used to replicate our approach in other groups (https://github.com/SethMusker/TargetVet).

## ﻿Introduction

The field of angiosperm phylogenetics has seen considerable advances in the last decade, much of which is owed to the democratisation of phylogenomics via target capture (Gnirke et al. 2009; Johnson et al. 2019; Zuntini et al. 2024). Target capture enables the production of phylogenomic datasets that are informative and universal at a fraction of the cost and effort required to generate equivalent data from whole genomes. It does so by deriving a customised subsample of the genome *in vitro* prior to sequencing. In principle, any given genomic region can be targeted as long as its sequence is known or can be approximated to within a minimum threshold of similarity (typically 70–80% sequence identity; Gnirke et al. 2009), which makes the method highly flexible and widely applicable. However, the flexibility of target capture presents researchers with a surprisingly complex challenge: deciding which genomic regions to target. Phylogenomic target gene sets have been designed to apply more or less universally across flowering plants, including the “Angiosperms353” gene set (Johnson et al. 2019) and the “mostly single-copy” gene set identified by De Smet et al. (2013) which the software MarkerMiner (Chamala et al. 2015) is designed to identify for a given taxonomic group. Nevertheless, there are demonstrable benefits of refining and/or extending these for specific groups, including improvements in capture success and informativeness (e.g., Folk et al. 2015; Kadlec et al. 2017; Straub et al. 2020; Ufimov et al. 2022).

When starting a target capture-based phylogenomic project, researchers need to decide whether to use a universal target set or one that is taxon-specific. While universal sets allow comparability of sequence data among different studies in flowering plants (Baker et al. 2021), they might be insufficient to achieve good phylogenetic resolution at the scale of interest. This risk is inherent to universal target sets because highly conserved genes are by nature slow-evolving, and as a result are likely to have relatively few phylogenetically informative sites, especially in recently and/or rapidly diversified taxa (e.g., Jones et al. 2019). Taxon-specific sets may therefore be more appropriate for phylogenetically “difficult” groups but may need to be built “from scratch”.

Designing a custom target set affords the researcher the opportunity to choose targets optimised for their study system, which might include attempting to maximise informativeness, minimise the chance of downstream errors, or allow for comparability to other data sets. Further, there may be the possibility of using newly available resources, such as draft genomes or new software, to improve on pre-existing target sets. Several factors need consideration when designing a custom target set:

- **Paralogy.** Paralogs are genes or genomic regions that have undergone one or more duplications in the deep past (Fitch 1970), often as a result of whole-genome duplications, which have been common throughout angiosperm evolution (Soltis et al. 2015; Tank et al. 2015). Confusing paralogs for orthologs (single-copy genes) can lead to erroneous phylogenetic inferences (Fernández et al. 2020). On the other hand, target capture efficiency is improved for paralogs because a single set of baits (the synthesised RNA fragments that bind to target regions during library preparation) can in theory capture all gene copies (Gardner et al. 2021; Ufimov et al. 2022). Using paralogs for phylogenetics does, however, require each gene copy to be distinguished within each species and then correctly grouped across species, which is computationally intensive and potentially error-prone (Ufimov et al. 2022; Zhou et al. 2022).
- **Informativeness.** In general, phylogenetic information is greater in faster-evolving regions of the genome. A common result in target capture-based phylogenetics is that non-coding sequences—in the form of introns and intergenic regions—significantly improve resolution when incorporated into phylogenetic analyses (e.g., Folk et al. 2015; Jones et al. 2019; Bagley et al. 2020; Maurin et al. 2021; Thomas et al. 2021). This is not just because of an increase in the overall information content of the data, but also because having informative individual loci is crucial for accurate phylogeny inference in the presence of incomplete lineage sorting (ILS; Avise et al. 1987; Maddison 1997; Degnan and Rosenberg 2006, 2009). Unfortunately, target capture usually only partially recovers non-coding regions because standard exon-based targets can capture non-coding sequences within just a few hundred base pairs flanking the exons (Gnirke et al. 2009).
- **Divergence.** Although target capture is tolerant to 20–30% sequence divergence between baits and the true target sequence, targets designed using distantly related sequences or used to capture loci across a set of highly divergent taxa can result in low capture efficiency due to excessive sequence divergence and/or gene loss (e.g., Johnson et al. 2019). Poor capture efficiency is the primary concern regarding explicitly targeting non-coding or otherwise rapidly evolving sequences, though a few studies have attempted this, and reported good capture rates (e.g., Folk et al. 2015; Karin et al. 2019).
- **Cost.** Custom bait designs are priced based on the target “footprint”, i.e., the total size of the bait set required to capture the full set of targets. The final cost therefore depends on several factors, including the total length of all targets combined, sequence complexity and uniqueness, and tiling (the degree to which neighbouring baits overlap). In general, designing a cost-effective target set imposes a trade-off between including more short loci *versus* fewer long loci.

The genus *Erica* is notably large, comprising over 851 species distributed in Europe and Africa (Elliott et al. 2024; Oliver et al. 2024). However, most species (ca. 690) are confined to the Cape Floristic Region (CFR) of South Africa where they represent a single monophyletic group (Pirie et al. 2016). This “Cape” clade shows clear indications of accelerated diversification upon its arrival in the CFR via the Afrotemperate region (Pirie et al. 2019), with a crown age of 6.0–15.0 Ma and net diversification rates of 0.28–0.97 species. Ma^−1^, which is notably higher than in other CFR angiosperm radiations (Pirie et al. 2016). This diversification surge is responsible for the genus being easily the largest in the CFR (Manning and Goldblatt 2012) and means that studying its diversification could shed light on the causes of the region’s exceptional floristic diversity (Linder 2003). At the same time, however, rapid diversification makes it extremely difficult to recover robustly resolved phylogenetic hypotheses, a fact that is well illustrated by the high degree of topological uncertainty throughout the Cape *Erica* clade in the most recently published phylogeny of the genus (Pirie et al. 2024), which was inferred using a small number of commonly used plant phylogenetic markers including nuclear ribosomal and various chloroplast regions.

### ﻿Aims and objectives

We set out to design a novel target set that would enable accurate phylogenomic analysis of closely related *Erica* species, but which could also be used to study relationships at both higher levels (e.g., between African and European *Erica* species, or between genera within Ericaceae) and lower levels (e.g., between closely related taxa in species complexes, or between populations within species). We implemented a mixed approach incorporating both universal and taxon-specific loci, as well as a mixture of intron-containing and exon-only loci. This approach aimed to balance concerns about paralogy, informativeness, comparability, and cost.

Implementing the approach involved (1) refining a pre-existing target set by iteration; (2) adding more targets derived from several recently published high-quality *Rhododendron* genomes and by reference to the angiosperm-wide Angiosperms353 target set; and (3) producing and using new whole-genome shotgun (WGS) sequencing data from three *Erica* species to quality check the new targets and produce *Erica*-specific versions of most targets, including the full gene sequences (exons and introns). We present a new pipeline to distinguish and identify paralogs during both customized target design and sequence data curation.

Furthermore, we investigate the impacts of alternative target set design choices on downstream analyses. Firstly, we ask whether draft genomes and WGS reads can be used to predict the presence and paralogy of potential targets. Secondly, we investigate the effect of different target identification methods on the usefulness and quality of the targets. Lastly, we examine the costs and benefits of explicitly targeting intronic regions with emphasis on capture efficiency and phylogenetic informativeness.

## ﻿Materials and methods

### ﻿Overview

Our primary goal was to improve on the work of Kadlec et al. (2017), who derived a set of 132 targets for *Erica* phylogenomics from a single *Rhododendron* transcriptome (*R.scopulorum* Hutch.; Matasci et al. 2014). Since those authors had tested their target set by conducting a target capture and sequencing experiment on several *Erica* samples, we used those data to produce *Erica*-derived versions of their targets. Subsequent to that study several highly complete and well-annotated *Rhododendron* genomes were published, bringing their number from zero in 2017 to three by the end of 2020 (Zhang et al. 2017; Soza et al. 2019; Yang et al. 2020). We therefore used these genomes to identify additional candidate targets. For this, we used two complementary methods. Firstly, we used MarkerMiner (Chamala et al. 2015) to mine the new *Rhododendron* genomes for “mostly single-copy” loci (De Smet et al. 2013). Secondly, we searched for genes from the Angiosperms353 target set (Johnson et al. 2019) in the three *Rhododendron* genomes.

The marker identification steps produced a very large number of candidate loci which we filtered based on a variety of criteria. Notably, we were able to evaluate not only the presence of each gene in *Erica* but also its status as single copy. This was enabled by newly generated high-depth shotgun WGS data from three *Erica* species. We made further use of this WGS data by building a draft genome of *Ericacinerea* L. and, where possible, we used its scaffolds to produce *Erica*-derived “full gene” versions of the targets, i.e., including both exon and intron sequences.

Finally, we conducted a target capture experiment with 295 samples (mostly of Cape *Erica*, but also including many European *Erica* species and additional genera serving as outgroups) using the newly developed target set, which comprised a total of 303 genes. We used the data from this experiment to evaluate the quality of each target in terms of capture efficiency, rate of paralogy, and phylogenetic informativeness, and tested whether these differed between targets produced using different methods. Specifically, we tested for differences between targets (1) identified by refining the Kadlec et al. (2017) set, (2) new targets found by MarkerMiner, and (3) targets matching the Angiosperms353 set. We also tested whether the quality of “full gene” targets derived from the *Ericacinerea* genome was better than that of targets derived from *Rhododendron* transcriptomes.

We developed a user-friendly suite of open-source command-line tools, TargetVet, which can be used to aid in developing and assessing a target set. The source code and a detailed account of the tool’s functionality and usage, including example code, are available at https://github.com/SethMusker/TargetVet. A diagram illustrating TargetVet’s functionality is presented in Suppl. material 1: fig. S1, with pertinent details provided in the following sections. The scripts are written in bash and R (R Core Team 2021).

### ﻿Whole-genome shotgun sequencing and assembly

Genomic DNA was extracted from fresh leaf material of three *Erica* species growing in the University of Bergen (UiB; Norway) arboretum following a custom protocol (Musker et al. 2024). These were (1) *E.cinerea* L. which is widespread across western Europe; (2) *E.trimera* (Engl.) Beentje from the East African highlands; and (3) *E.cerinthoides* L. which is widespread in the CFR and further east in South Africa. Library preparation and sequencing was conducted by the Genomics Core Facility at UiB. Sequencing was done using a single Illumina NovaSeq 6000 SP flowcell to generate 2 × 150 bp paired-end reads.

Raw reads were trimmed using fastp (Chen et al. 2018) followed by deduplication using clumpify.sh from BBTools v.38.90 (BBMap – Bushnell B. – https://sourceforge.net/projects/bbmap/). Overlapping read pairs were merged using bbmerge-auto.sh from BBTools, keeping un-merged pairs. Read quality was checked with FastQC (https://www.bioinformatics.bbsrc.ac.uk/projects/fastqc) and MultiQC (Ewels et al. 2016). Draft genomes were assembled using ABySS v.2.2.5 (Simpson et al. 2009; Jackman et al. 2017) using both merged and un-merged reads. Assembly statistics such as N50 and L50 were calculated by ABySS and BBToolsstats.sh. To further assess genome completeness on the basis of gene recovery, we used BUSCO v.5.0.0 (Simão et al. 2015). BUSCO searches the assembly for genes that are confidently thought to be single-copy and reports completeness- and duplication-related statistics. We ran BUSCO separately for each assembly with the same parameters: Reference universal single-copy orthologs were from the “eudicots_odb10” lineage dataset version 2020-09-10, which consists of 2326 genes from 31 species, and metaeuk v.4 (Karin et al. 2020) was used as the gene predictor. BUSCO results were summarised using the bundled script generate_plot.py, which uses ggplot2 (Wickham 2016).

### ﻿Designing a target set for *Erica* phylogenomics

#### ﻿Refining the Kadlec et al. (2017) target set

Refinement method

Kadlec et al. (2017) conducted their target capture experiment using 25 species of Cape *Erica*. Because a major objective of our broader project was to resolve relationships in the *E.abietina*/*E.viscaria* clade (Pirie et al. 2017), we retrieved the reads from the single sample of *E.grandiflora* – the only member of that clade in the sample set – and used HybPiper v.1.3.1 (Johnson et al. 2016) to assemble the 134 targets of Kadlec et al. (2017) (132 nuclear loci identified by MarkerMiner plus two “universal” loci, rpb2 and topoisomerase B). Additional programs used by HybPiper were BWA-MEM v.0.7.17-r1188 (Li 2013) for read mapping, SPAdes v.3.13.0 (Bankevich et al. 2012) for contig assembly, and exonerate v.2.2.0 (Slater and Birney 2005) for identifying exon-intron boundaries. We then based our new targets on the 134 assembled supercontigs (i.e., scaffolds including flanking regions, exons, and introns). In order to avoid targeting poorly recovered genes, if a supercontig’s length was less than 70% of the length of its corresponding *Rhododendron* CDS the latter was taken instead. It is important to note that this filter was agnostic to the make-up of the supercontigs. For example, supercontigs with missing exons could still be included.

#### ﻿Identifying new targets

##### MarkerMiner method

We used MarkerMiner v.1.2 (Chamala et al. 2015) to search for putative single-copy orthologs from the gene set identified by De Smet et al. (2013). We used the *Vitisvinifera* single-copy reference genes, setting the minimum transcript length to 900 bp. Three *Rhododendron* CDS files were used to find matches: (1) *R.simsii* Planch. (Genbank: ASM1428224v1, accessed 02.11.2020 Yang et al. 2020), (2) *R.williamsianum* Rehder & E.H.Wilson (Genbank: ASM974610v1, accessed 02.11.2020 Soza et al. 2019), and (3) R.delavayiiFranch.var.delavayi (http://dx.doi.org/10.5524/100331, accessed 02.11.2020; Zhang et al. 2017). We employed three initial filters on this set. Firstly, we discarded genes not present in both *R.simsii* and *R.delavayii*. Presence in *R.williamsianum* was not included as a filtering criterion because it returned relatively few hits (Suppl. material 1: fig. S2). Secondly, we kept only the longest sequence out of the three potential *Rhododendron* targets. Lastly, we used BLASTn (e-value: 1e^−5^, BLAST v.2.10.1+; Altschul et al. 1997) to identify targets already present in the Refinement set and removed them if there was at least one match.

Because MarkerMiner identified many more genes than could be added to the target set given the total footprint available to the project (Suppl. material 1: fig. S2), we implemented a pre-filtering step for the MarkerMiner genes prior to further filtering. As off-target reads from target capture experiments are essentially equivalent to shotgun reads (Costa et al. 2021), we used the off-target reads from the Kadlec et al. (2017) experiment to identify the MarkerMiner genes that were most likely to be present in *Erica*. Reads were pooled across the *Erica* samples (n = 25) in the Kadlec et al. (2017) data and mapped to the MarkerMiner genes with NextGenMap v.0.5.5 (Sedlazeck et al. 2013). We chose to use NextGenMap because it tolerates greater levels of sequence divergence than BWA-MEM (Sedlazeck et al. 2013), which was useful given that the number of off-target reads was relatively small. Depth per position was determined using BamTools v.2.1.1 (Barnett et al. 2011) and the mean depth was calculated as the total depth divided by the gene’s length. We discarded genes not having at least 80% of their length covered by at least one read. Of those, we kept genes with depth greater than—but still within two standard deviations of—the “grand” mean depth (i.e., across all genes). Finally, we discarded genes < 1,500 bp long.

##### NewTargets method

To incorporate the widely used Angiosperms353 target set, we adapted “NewTargets” developed by McLay et al. (2021, https://github.com/chrisjackson-pellicle/NewTargets) to the task of finding *Rhododendron* genes matching the Angiosperms353 targets. We used the script BYO_transcriptome.py to search for *Rhododendron* versions of the Angiosperms353 genes. The “Mega353” gene set, an expanded Angiosperms353 set with many additional taxa representing each sequence (McLay et al. 2021), was used as the reference. The three *Rhododendron* CDS files (see above, MarkerMiner method) were used as the input transcriptomes. To identify homologous sequences in the transcriptomes, BYO_transcriptome.py uses hidden Markov model profiles of the reference genes made with HMMER3 (Mistry et al. 2013). The chosen settings disabled grafting to prevent the formation of chimeric sequences (-no_n) and discarded transcripts whose length was < 70% that of the mean of the reference sequence homolog (-discard_short -length_percentage 0.7). We extracted the longest of the three potential *Rhododendron* targets and discarded those shorter than 1,000 bp. We used BLASTn as before to identify and remove any targets already present in the MarkerMiner or Refinement sets.

##### Filtering the target sets using WGS reads

Because WGS sequencing represents a largely unbiased method of deriving sequences from a genome, we reasoned that read mapping depth information could be used to infer (1) presence/absence and (2) paralogy of the candidate targets in *Erica*. In theory, missing targets should have a depth of zero while duplicated regions should have a depth roughly twice that of the mean across all targets (assuming most targets are single-copy). *Ericacinerea* has a considerably smaller genome than most *Erica* species with genome size data, including *E.trimera* (based on the assembly size) and *E.cerinthoides* (Mugrabi De Kuppler 2013), which may indicate a lower rate of paralogy and/or more missing genes, though could also be due to lower repetitive DNA content. We therefore excluded *E.cinerea* from the next step, in which we mapped the WGS reads from *E.trimera* and *E.cerinthoides* separately to the potential targets using BWA-MEM v.0.7.17 with default parameters, then used SAMtools v.1.11 (Danecek et al. 2021) to keep only hits with mapping quality > 20, and finally calculated read depth at each position using BamTools. We removed any target whose median depth deviated by more than one standard deviation from the mean depth across all targets for either of the two *Erica* species. This process was repeated for each target set separately (Refinement, MarkerMiner, and NewTargets).

Additionally, for the Refinement set we applied the above process separately to the *E.grandiflora*-derived supercontigs and the original transcript-derived targets and added the latter to the final set if they passed the filters but the former failed. We added to TargetVet a pair of command-line scripts (map_WGS_to_targets.sh and VetTargets_WGS.R) which can be applied to any data when provided with one or more WGS read files and a set of target sequences (Suppl. material 1: fig. S1).

#### ﻿Extracting *Erica*-derived targets

We next aimed to produce *Erica*-derived versions of the new MarkerMiner and NewTargets sets, with the aim being to improve capture efficiency by increasing sequence similarity and including introns. We chose to use only the *E.cinerea* assembly as it was by far the most contiguous and complete of the three. We removed any scaffolds in the assembly < 500 bp long. The targets were translated to protein sequences using EMBOSS (Madeira et al. 2022) and these were then mapped to the *E.cinerea* draft genome assembly using tBLASTn (adding the option -max_target_seqs 50000 to ensure that all matches were returned; Shah et al. 2019). We kept matches with sequence identity ≥ 70% and E-value < 1e^−6^, and only kept targets if > 70% of their length mapped to a single *E.cinerea* scaffold (i.e., discarding any that mapped to more than one scaffold). We calculated the length of the mapped region in the *E.cinerea* genome as the difference between the largest end position and the smallest start position of the blast matches, giving an estimate of the total gene length including exons and introns. We extracted these genomic sequences using Rsamtools v.2.10.0 (Morgan et al. 2021). This process was automated within TargetVet as an R script: TargetSupercontigs.R.

The WGS read depth-based filtering procedure described above was repeated for the genomic sequences to help ensure that they were present and single-copy across their full length in other *Erica* species. Genomic sequences that failed read depth filtering were reverted to their *Rhododendron* transcript version (which had already passed the filters), while those that passed were substituted in for their corresponding *Rhododendron* transcripts.

#### ﻿Evaluating the target set’s quality

##### Sequencing new samples

The final target set was used in a target capture experiment including 295 samples, mostly of Cape *Erica* species. DNA was extracted using a custom protocol (Musker et al. 2024). Bait design (3X tiling), bait synthesis, library preparation and sequencing were carried out by Daicel Arbor BioSciences (Ann Arbor, MI 48103, United States). Samples were paired-end sequenced using an Illumina NovaSeq 600 instrument to 2 × 150 bp. To quality-filter, trim and deduplicate the raw reads we used fastp v.0.23.2 (parameters: –detect_adapter_for_pe –dedup –overrepresentation_analysis –trim_poly_g –qualified_quality_phred 20 –unqualified_percent_limit 30 –average_qual 20 –length_required 100).

##### Target assembly

To investigate the effects of target source (i.e., *Rhododendron* CDS *versus Erica* genome) and marker identification method (i.e., Refinement, MarkerMiner and NewTargets) on aspects of target recovery and assembly, we assembled the targets from all 295 samples using HybPiper v.2.0.1. We ran HybPiper’s assemble module using BWA-MEM v.0.7.17 for read mapping, SPAdes v.3.15.3 for assembly (with kmer values of 33 and 77), exonerate v.2.4.0, and BBTools v.38.92.

Prior to assembly with HybPiper, in order to ease computational burden we used reformat.sh from BBTools to randomly subsample each sample’s reads to one million read pairs. Given a total target footprint of 1,161,538 bp and assuming a mean read pair length of ca. 290 bp (to account for trimming and pair overlaps), this gives an expected mean coverage of ca. 250X.

##### Assessing paralogy and missingness

**Figure 1.**
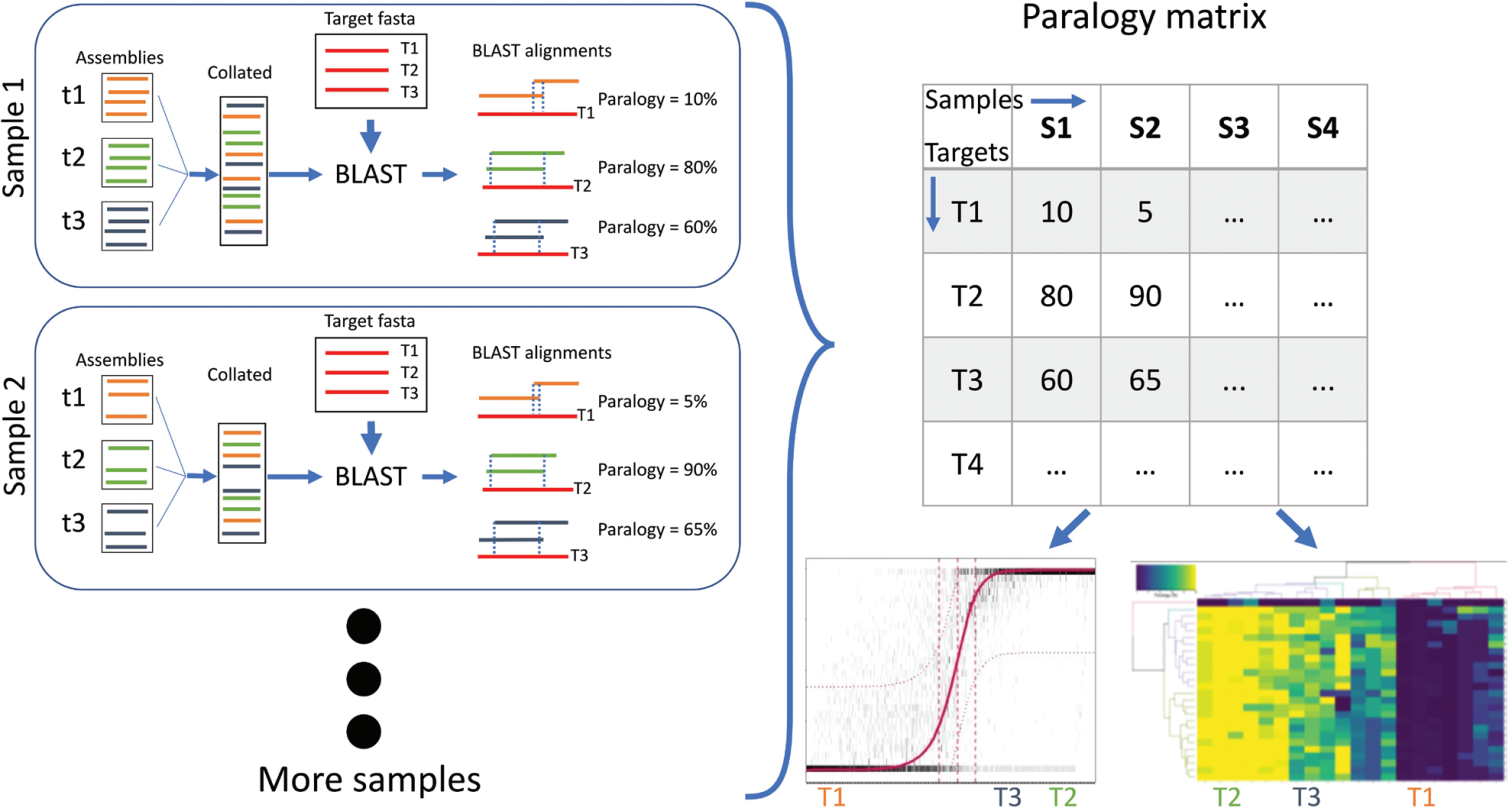
Graphical illustration of how TargetVet’s VetHybPiper.sh script estimates paralogy from HybPiper results. First, the assemblies for each target are collated into a single multifasta. These scaffolds are then matched to the reference targets using BLAST. Using the BLAST result, VetTargets_genome.R calculates paralogy % for each target. This process is repeated for each sample in order to populate the paralogy matrix, which DetectParalogs.R analyses to produce summary statistics and visualisations.

To investigate paralogy we first used HybPiper’s length-based criterion which, on a per-sample basis, flags a target as a potential paralog if its second-longest contig’s length is above a certain proportion (which we set to 0.75, the default) of the longest contig’s length (Johnson et al. 2016). Secondly, we developed a custom coverage-based approach which characterises paralogy and flags putative paralogs based on information across the full sample set. We incorporated the approach into a command-line utility in the form of a bash script (VetHybPiper.sh), which acts largely as a wrapper around BLAST and several custom R scripts that are part of TargetVet (Suppl. material 1: fig. S1). A graphical illustration of the method is provided in Fig. 1, and it proceeds as follows:

1. For each sample,

1. map all assembled contigs to the target sequences using BLAST;
2. remove matches below given thresholds of length (by default, 150 bp) and sequence similarity (by default, 70%);
3. for each target, calculate each site’s coverage (*c*) by counting how many BLAST matches from different contigs map to it;
4. define *L* as the total length of the target in base pairs (i.e., number of sites) and *l _c_* as the number of sites with coverage = *c*;
5. estimate each target’s paralogy (*P*) as the fraction of its length with *c* ≥ 2, ignoring missing regions, i.e.,

$P=\frac{l_{c\geq 2}}{L-l_{0}}$.

1. Across all samples, flag targets as putative paralogs if *P* is unusually high compared to most targets.

Additionally, using the above definitions, missingness (*M*) can be estimated as the fraction of the target’s length with *c* = 0, and copy number (*C*) can be estimated as the mean coverage across sites ignoring sites with *c* = 0.

Estimates of *P*, *M* and *C* were derived from two separate BLASTn mapping results: one in which the actual target sequences were used as the reference, and one in which the transcript versions of the targets were used as the reference. To remove putative paralogs, we discarded targets with mean *P* (across 295 samples) > 40% according to either of the two BLAST results (n = 13). To remove targets that were poorly recovered, we discarded those with mean *M* > 40% according to the BLAST result based on the target sequences (n = 5). This reduced the total number of genes from 303 to 285, and herein we refer to these target sets as “Erica303” and “Erica285”, respectively. Unless otherwise stated, all further analyses used the Erica285 target set.

##### Assessing target and intron capture efficiency

To test whether *Erica* genome-derived targets had greater capture efficiency than *Rhododendron* CDS-derived targets, we used separate fixed effect models for each marker identification method to model supercontig length as a function of target source, including sample as a fixed effect to account for random variance, while also allowing the sample effect to vary by transcript length to account for the tendency for longer transcripts to have longer supercontigs. We used HybPiper’s stats module to collect transcript and supercontig lengths for all samples.

Exon-derived baits are only able to capture intronic sequences flanking the exons, meaning that sequence coverage drops off considerably with increasing distance from the nearest exon (Gnirke et al. 2009), such that long introns are often not fully recovered. We therefore hypothesised that, because they included intronic sequences, *Erica* genome-derived targets would recover more complete introns than *Rhododendron* CDS-derived targets, but only when introns were long enough to fail to be caught by exon-derived baits. Specifically, we predicted that as total gene length increased, CDS-derived targets would exhibit an obvious “drop-off” in recovered intron length beyond a certain point, whereas genome-derived targets would show a steady increase in intron length with increasing total gene length. To test this prediction, we determined the total length of intronic sequence assembled for each gene for each sample using the annotations from exonerate’s protein2genome model, setting the intron length to zero if no intronic region was identified. We used separate fixed effects linear models for each target identification method to model intron length as a function of gene length and target source, including sample as a fixed effect. We included the source by gene length interaction term to test whether the slope of the relationship between gene length and intron length was significantly lower for CDS-targeted genes, as per our prediction. As a proxy for the gene’s “true” length we used the maximum gene length (across all samples) inferred by exonerate. This was likely to be an underestimate for many CDS-targeted genes, especially longer genes whose full intronic sequence may not have been recovered in any sample, meaning that estimated differences in slope were likely to underestimate the true difference. Models and significance tests were run using fixest (Bergé 2018).

#### ﻿Evaluating the target set’s phylogenetic utility

To assess the usefulness of the targets for phylogenomics, we selected a subset of 32 samples including three outgroup samples (*Calluna*, *Daboecia*, and *Rhododendron*) and eight European, one Madagascan, one East African, and 19 Cape *Erica* (details in Suppl. material 1: table S2). We aimed to characterise the ability of the target sets to (1) recover well-established relationships based on previous work, and (2) resolve relationships between Cape *Erica* clades that have shown evidence of recent and rapid diversification (Pirie et al. 2011, 2016). We investigated how these properties were affected by the presence or absence of paralogs or largely missing targets (Erica303 *versus* Erica285), as well as target source (*Rhododendron* CDS *versus Erica* genome) and marker identification method (Refinement, MarkerMiner and NewTargets). We restricted the analyses to supercontig sequences in order to maximise sequence length and thus variation (Bagley et al. 2020).

##### Multiple sequence alignment

Supercontig MSAs were generated using the L-INS-i algorithm of MAFFT (Katoh and Standley 2013), after which poorly aligned ends of individual sequences were recoded as missing using a custom modification of HerbChomper (Gardner 2021), a fork of the HerbChomper tool available at github.com/SethMusker/HerbChomper_MSA. The original HerbChomper algorithm takes a user-specified sequence in an MSA (the “reference”) and calculates sequence identity between the reference and another user-specified sequence (the “target”) along a sliding window of a given number of nucleotides, with two rounds (forward and reverse) each of which starts from one end of the alignment and works inwards. Each round recodes as gaps (“-”) any target nucleotides that fall within a window whose sequence identity (relative to the reference sequence in that window) falls below a given threshold and stops when the sequence identity of a window reaches the threshold. The modified implementation calculates the majority-rule consensus of the alignment using seqinr (Charif and Lobry 2007) and uses that as the reference sequence to recode each individual sequence in the alignment separately. We used a sliding window of 50 bp and a sequence identity threshold of 0.8 for all MSAs. Finally, gappy regions of the MSAs were removed using ClipKIT smart-gap (Steenwyk et al. 2020), which aims to remove gappy regions without introducing potential errors caused by excessive trimming (Tan et al. 2015).

#### ﻿Species tree concordance

##### Species tree inference

Species trees were estimated using a concatenation method and a summary coalescent method. For the concatenation method, IQ-TREE v.2.2.0 (Minh et al. 2020) was used with an edge-linked proportional partition scheme, setting each target as a separate initial partition. ModelFinder (Kalyaanamoorthy et al. 2017) was used for substitution model estimation and partition merging (to reduce over-fitting) while only examining the top 25% of partitioning schemes (Lanfear et al. 2014) to reduce computational burden. Branch support values were estimated using ultrafast bootstrap (UFBoot; Hoang et al. 2018) and SH-alrt (Guindon et al. 2010) with 1,000 replicates each.

For the summary coalescent method we used a modification of ASTRAL (Zhang et al. 2018), Weighted ASTRAL – Hybrid (wASTRAL–h) v.1.8.2.3 (Zhang and Mirarab 2022), which weights quartets by both branch length and local support values to provide more accurate species tree inferences than the unweighted ASTRAL algorithm. Gene trees were estimated by maximum-likelihood (ML) using IQ-TREE with two independent runs to improve the tree search after automated substitution model selection using ModelFinder, with UFBoot (1,000 replicates) used to estimate branch support. We ran wASTRAL–h with the flag “–moreround” to increase the number of placement and subsampling rounds from four to 16 for a more thorough search of the tree space. Herein we refer to wASTRAL–h simply as ASTRAL.

As a means of assessing the impact of paralogs and poorly recovered loci on phylogenetic inference, we ran both IQ-TREE and ASTRAL analyses separately on the Erica303 and Erica285 target sets.

##### Topological concordance

We compared trees inferred using different marker sets and different methods using cophylo from phytools (Revell 2011). To assess the results in the context of previous work, we also compared the newly inferred trees to the most recent *Erica*-wide phylogeny (Pirie et al. 2024), which was inferred based on ribosomal and chloroplast markers using RAxML v.8.0.0 (Stamatakis 2014) with standard non-parametric bootstrapping (100 replicates) and originally included 752 tips. We trimmed the tree to include only the species or subspecies shared between the sample sets (n = 30) using the ape function drop.tips.

##### Phylogenetic informativeness

Lastly, we aimed to investigate the effects of marker identification method and target source on phylogenetic informativeness. AMAS (Borowiec 2016) was used to determine the number of parsimony-informative sites in each alignment. PhyInformR (Dornburg et al. 2016) was used to estimate Quartet Internode Resolution Probability (QIRP), which is a measure of phylogenetic informativeness that accounts for sequence substitution rate variation, tree depth, and internode length.

We estimated QIRP for the crown of the clade consisting of the *E.abietina*/*E.viscaria* clade, the *E.massonii* clade, and the *E.corifolia* clade. All of these clades were recovered with good support by Pirie et al. (2016). We refer to this as the “VMC clade”, and chose to focus on it due to (1) its young crown age (ca. 5 Ma; Pirie et al. 2016) and (2) the very short internodal branches separating the three crowns of the constituent sub-clades (all < ca. 1 million years; Pirie et al. 2016). We estimated an ultrametric tree (as required by PhyInformR) based on the concatenation phylogeny using chronos in ape (Paradis 2013; Paradis and Schliep 2019). We estimated site substitution rates using IQ-TREE v.2.2.0 (Minh et al. 2020), using the empirical Bayesian method and the best model and partition-merging scheme as estimated for the concatenation-based phylogenetic analysis.

## ﻿Results

### ﻿Genome assembly results

**Figure 2.**
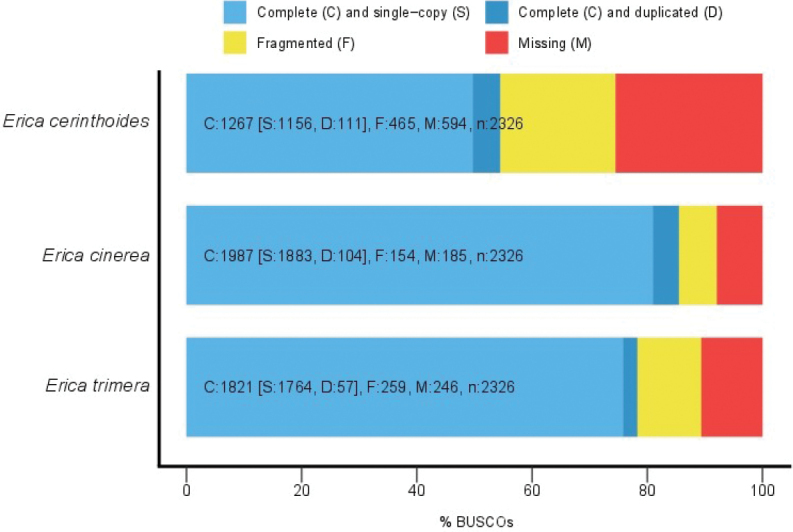
Graphical summary of the BUSCO results for the three assembled *Erica* draft genomes. Despite their fragmented nature, the genomes have reasonably good gene recovery rates.

The quality of the draft genome assemblies of *Ericacinerea*, *E.trimera*, and *E.cerinthoides* varied considerably (Table 1; Fig. 2). The much greater contiguity of the *E.cinerea* assembly compared to that of the other species was most notable. This was most likely a result its much smaller genome size as approximated by the total sequence length of the assemblies (Table 1), combined with the sample having ca. 20% more reads. The *E.cinerea* assembly also had much better completeness based on the BUSCO results, likely due to its greater contiguity. The low proportions of duplicated BUSCOs suggest that the three species are all diploid. Overall, the assemblies are of reasonable quality and should prove useful for genomic studies in *Erica* beyond the present work.

**Table 1.** Assembly statistics of the three newly assembled *Erica* draft genomes.

	*E.cinerea*	*E.trimera*	*E.cerinthoides*
Read statistics			
Number of read pairs	340,904,000	282,465,000	284,039,000
% reads merged	50.69%	43.84%	43.97%
Mean insert size	306.8 bp	299.1 bp	303.4 bp
Assembly statistics			
Scaffold sequence total	353.050 Mb	708.005 Mb	679.014 Mb
Number of scaffolds	286,992	1,852,782	1,463,182
Number of scaffolds > 50 kb	670	51	1
% genome in scaffolds > 50 kb	13.11%	0.43%	0.01%
Scaffold N50	5,597	124,874	73,631
Scaffold L50	15,727 bp	616 bp	1,028 bp
Max. scaffold length	192,106 bp	121,715 bp	54,438 bp
Mean (SD) GC content	39.5% (0.92%)	44.9% (1.08%)	40.3% (0.89%)

### ﻿Target set design results

#### ﻿Refinement method

Of the 134 Kadlec et al. (2017) targets, two were found to be almost identical (sequence similarity = 99.8%, identical length), so one of them was arbitrarily discarded. *Ericagrandiflora* supercontigs were assembled for all targets, of which 92 passed the WGS depth-based filtering. Of the remaining targets, the transcript sequence of a further 13 passed the filtering, bringing the total number of targets in the Refinement set to 105.

#### ﻿MarkerMiner method

A total of 1,572 mostly single-copy genes were identified by MarkerMiner as being present in at least one of the three *Rhododendron* transcriptomes (Suppl. material 1: fig. S2). Of these, 1,293, 1,217 and 999 were present in *R.simsii*, *R.delavayi*, and *R.williamsianum*, respectively. Of the 1,021 genes present in both *R.simsii* and *R.delavayi*, 16 were discarded as they had significant BLAST hits to Kadlec et al. (2017) targets. The pre-filtering step based on off-target read depth and sequence length (≥ 1,500 bp) reduced the number of genes from 1,005 to 129, while the WGS depth-based filtering further reduced the set to 114 genes. A total of 71 of these genes had good matches in the *E.cinerea* genome, all of which passed depth-based filtering. This left 43 genes represented by their transcript sequence in the final MarkerMiner set.

#### ﻿NewTargets method

Of the 353 genes in the Mega353 reference set, 348 were found in at least one of the three *Rhododendron* transcriptomes and 101 of these were longer than 1,000 bp. Of these, 87 passed WGS depth-based filtering, 59 of which had good matches in the *E.cinerea* genome. Seven of these failed depth-based filtering and were reverted to their transcript form, leaving 52 genomic sequences and 35 transcript sequences in the final NewTargets set.

#### ﻿Combined target superset

After all of the above steps the final combined target “superset” consisted of 303 targets with a combined length of 1,161,538 bp, which we refer to as the “Erica303” set.

### ﻿Target capture experiment results

#### ﻿Paralogy

**Figure 3.**
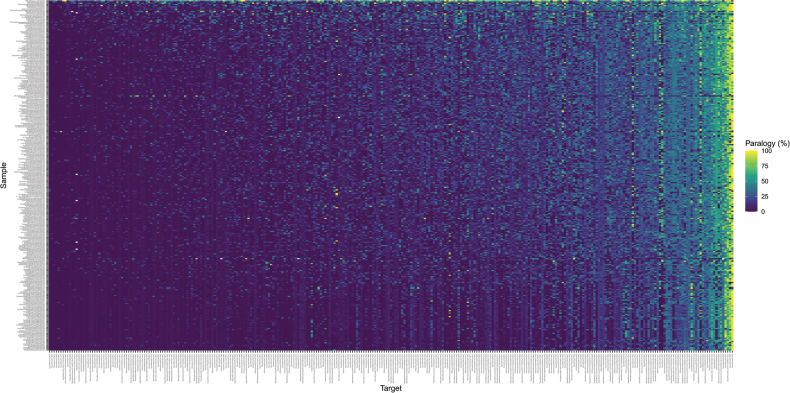
Heatmap showing paralogy (*P*), the estimated proportion of a target’s length covered by more than one assembled contig, for all samples and all loci in the Erica303 superset. Values of *P* were calculated from BLAST results using the actual target sequences. Targets and samples are arranged by mean *P.* This plot is a direct product of the TargetVet script VetHybPiper.sh.

Overall paralogy was low across the target superset according to both length- and coverage-based analyses (Suppl. material 1: fig. S3 and Fig. 3, respectively), although the length-based method was apparently less sensitive. These results suggest that the WGS depth-based filtering method was largely successful in identifying paralogs. *P* was largely unaffected by whether it was estimated using the actual targets or their CDS versions (Suppl. material 1: fig. S4), with the exception of two Refinement targets that had high CDS-based *P* but low target-based *P*.

**Figure 4.**
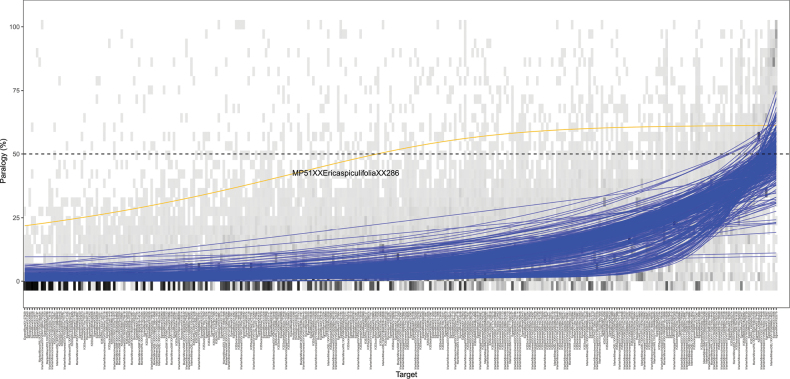
Patterns of paralogy (*P*) per sample. Targets (x-axis) are arranged in ascending order by mean *P* across all samples. Curves show the predicted *P* for each sample obtained from n-parameter logistic regressions. The single sample that deviated from the mean *P* by more than 20% on average across all targets is highlighted (yellow line) and labelled. This plot is a direct product of the TargetVet script VetHybPiper.sh.

Most samples showed similar paralogy patterns (Fig. 4), with the notable exception of the single *Ericaspiculifolia* sample, which had a mean *P* of 47.0% (27.3% SD), 142 targets with *P* > 50%, and a mean copy number (*C*) of 1.65 (0.491 SD). *Ericaspiculifolia* has a 1.5-fold higher chromosome number (2n = 36) than most *Erica*, which typically have 2n = 24 (Nelson and Oliver 2005), making ploidy the most likely explanation for this finding.

#### ﻿Target recovery

Genome-derived targets produced significantly longer supercontigs than CDS-derived targets for the MarkerMiner (1,162 bp longer) and NewTargets (1,647 bp longer) sets, but significantly shorter supercontigs for the Refinement set (1,075 bp shorter; Suppl. material 1: table S1). Nevertheless, *R*^2^ values were generally low even when accounting for variance explained by CDS length and sample identity (highest *R*^2^ = 0.264, highest within-*R*^2^ = 0.077), suggesting that variation in supercontig length was not well-predicted. This was most likely because supercontig length was not primarily determined by CDS length but rather by true target length (i.e., including introns), which could not be modelled because true target lengths were unknown for the CDS-derived targets. Nevertheless, the significantly shorter CDS-derived supercontigs in the MarkerMiner and NewTargets sets illustrate the benefits of using genome-derived targets.

#### ﻿Intron recovery

**Figure 5.**
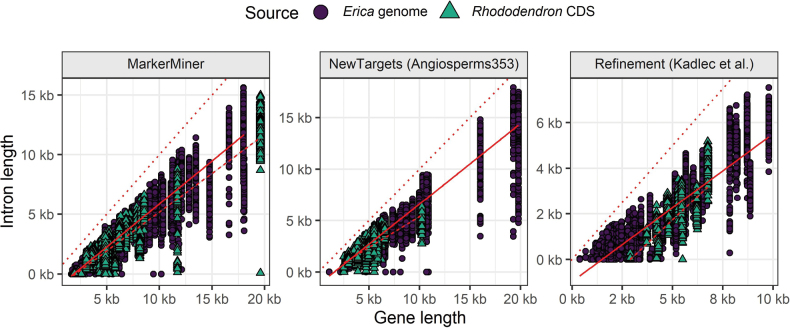
The relationship between gene length and intron length depends on the source of the target and the method of target set design. For MarkerMiner and NewTargets targets, the slope is steeper for genome-derived targets (solid lines) than for CDS-derived targets (dashed lines). For Refinement targets, the slope is steeper for CDS-derived targets, though these also have relatively less intronic sequence on average. The dotted lines indicate the 1:1 line. Results of the statistical tests to compare the slopes are given in Table 2.

The analysis of intron length in relation to gene length suggested that *Erica*-derived targets captured relatively more intronic sequence (Table 2, Fig. 5). Specifically, for the MarkerMiner and NewTargets sets intron length increased with gene length more steeply for the genome-derived target sets (MarkerMiner: slope = 0.721, NewTargets: slope = 0.781) than for the CDS-derived sets (MarkerMiner: slope = 0.650, NewTargets: slope = 0.598). For the Refinement set the slope difference was reversed (CDS-derived: slope = 0.826, genome-derived: slope = 0.648), however, the intercept difference estimate showed that the CDS-derived supercontigs had, on average, less intronic sequence than the genome-derived supercontigs (Fig. 5). While it is possible that sequence similarity could explain these results (i.e., *Erica*-derived baits capture *Erica* DNA more effectively than *Rhododendron*-derived baits), the high capture efficiency of the CDS-derived baits (Suppl. material 1: table S1) suggests that target capture was not hampered by sequence divergence. Rather, the results supported the hypothesis that explicitly targeting introns results in improved intron recovery by mitigating the decline in capture efficiency further from exons.

**Table 2.** Results of the fixed effects models of intron length as a function of target source and gene length, showing that longer introns were recovered by *Erica* genome-derived targets identified using NewTargets and MarkerMiner, whereas longer introns were recovered by *Rhododendron* CDS-derived targets identified using the Refinement method. The relationship was unaffected by sample identity (*R*^2^ ≈ Within *R*^2^). Numbers in brackets are standard errors.

	MarkerMiner	NewTargets	Refinement
Gene length × Source = *Erica* genome: slope	0.721***	0.781***	0.648***
	(0.003)	(0.005)	(0.002)
Gene length × Source = *Rhododendron* CDS: slope	0.650***	0.598***	0.826***
	(0.005)	(0.003)	(0.005)
Source = *Rhododendron* CDS: intercept	18.0	607.2***	-1,428.7***
	(21.1)	(27.2)	(18.4)
Observations	33,599	24,691	30,957
R2	0.900	0.904	0.795
Within R^2^	0.900	0.904	0.795

Signif. codes: *** = 0.01, ** = 0.05, * = 0.10.

### ﻿Species tree concordance

**Figure 6.**
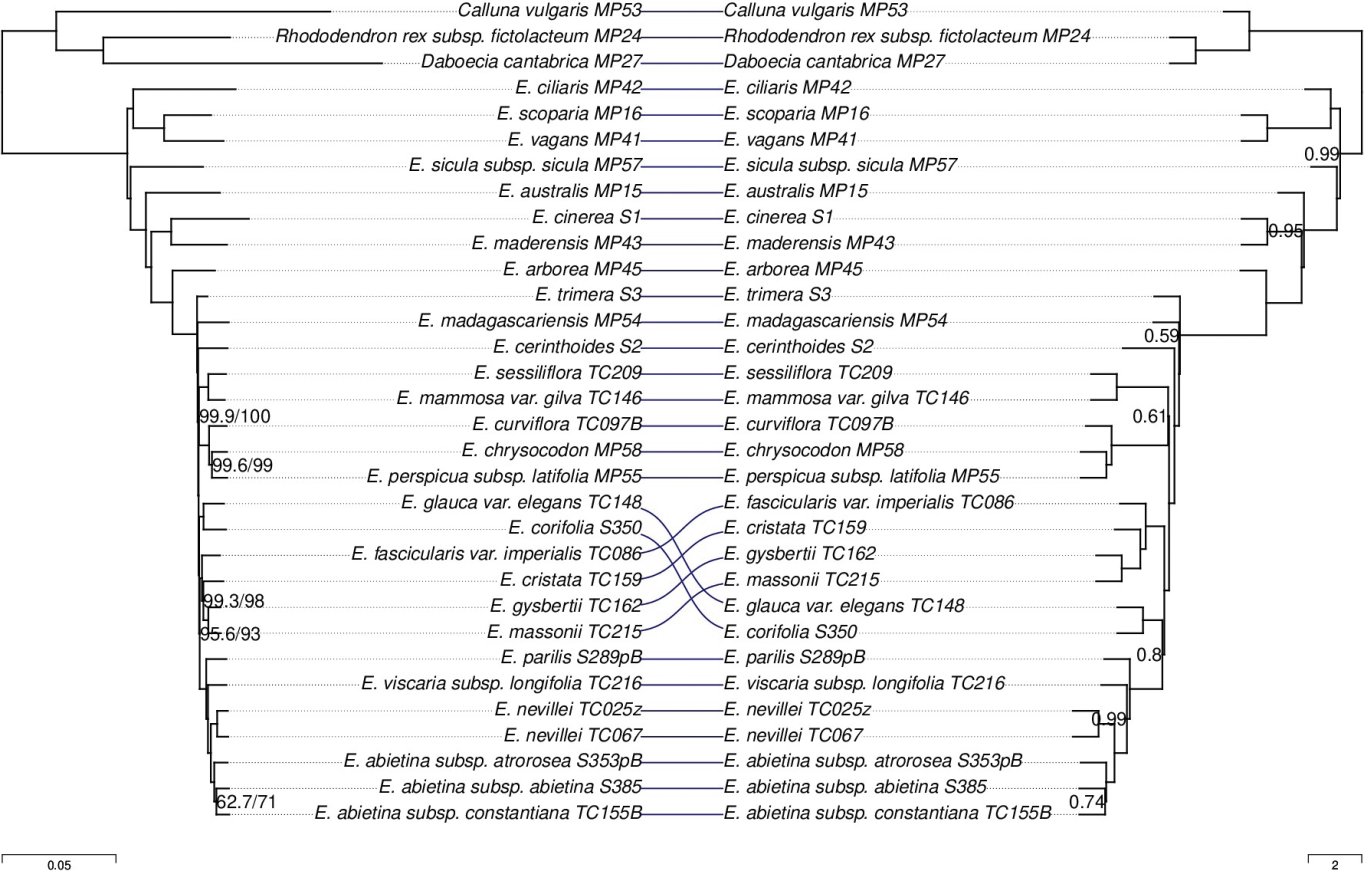
Tanglegram comparing the phylogenies inferred by concatenation (IQ-TREE; Left) and by ASTRAL (Right) using the Erica285 target superset, which excludes putative paralogs and genes with excessive missing data. For the concatenation tree, branch lengths are in substitutions per site and node labels are SH-alrt/UFBoot percentages. For the ASTRAL tree, branch lengths represent coalescent units (except for terminal branches which are are arbitrarily set to 1 as they are not estimated by ASTRAL) and node labels show posterior probability support. Nodes with full support are unlabelled. The trees are fully bifurcating and are rooted along the branch between the *Erica* and non-*Erica* samples arbitrarily for display purposes.

The presence of paralogs and poorly recovered genes had no effect on species tree topology and little effect on branch support (Suppl. material 1: fig. S5, Fig. 6). In contrast, the effect of phylogenetic reconstruction method was notable. In general, branch support values were higher in the concatenation trees than in the ASTRAL trees. Trees inferred using the two methods differed in the topology of the “VMC clade”: concatenation recovered the *E.corifolia* clade as sister to the *E.abietina*/*E.viscaria* and *E.massonii* clades, i.e., the topology (C,(M,V)), whereas ASTRAL recovered the topology (M,(C,V)). However, this resolution had relatively low local posterior probability (PP = 0.8) in the ASTRAL trees (Suppl. material 1: fig. S5, Fig. 6) and low support (SH-alrt/UFBoot = 86/86) in the concatenation tree based on the Erica303 set (Suppl. material 1: fig. S5), and therefore the conflict was not strongly supported.

**Figure 7.**
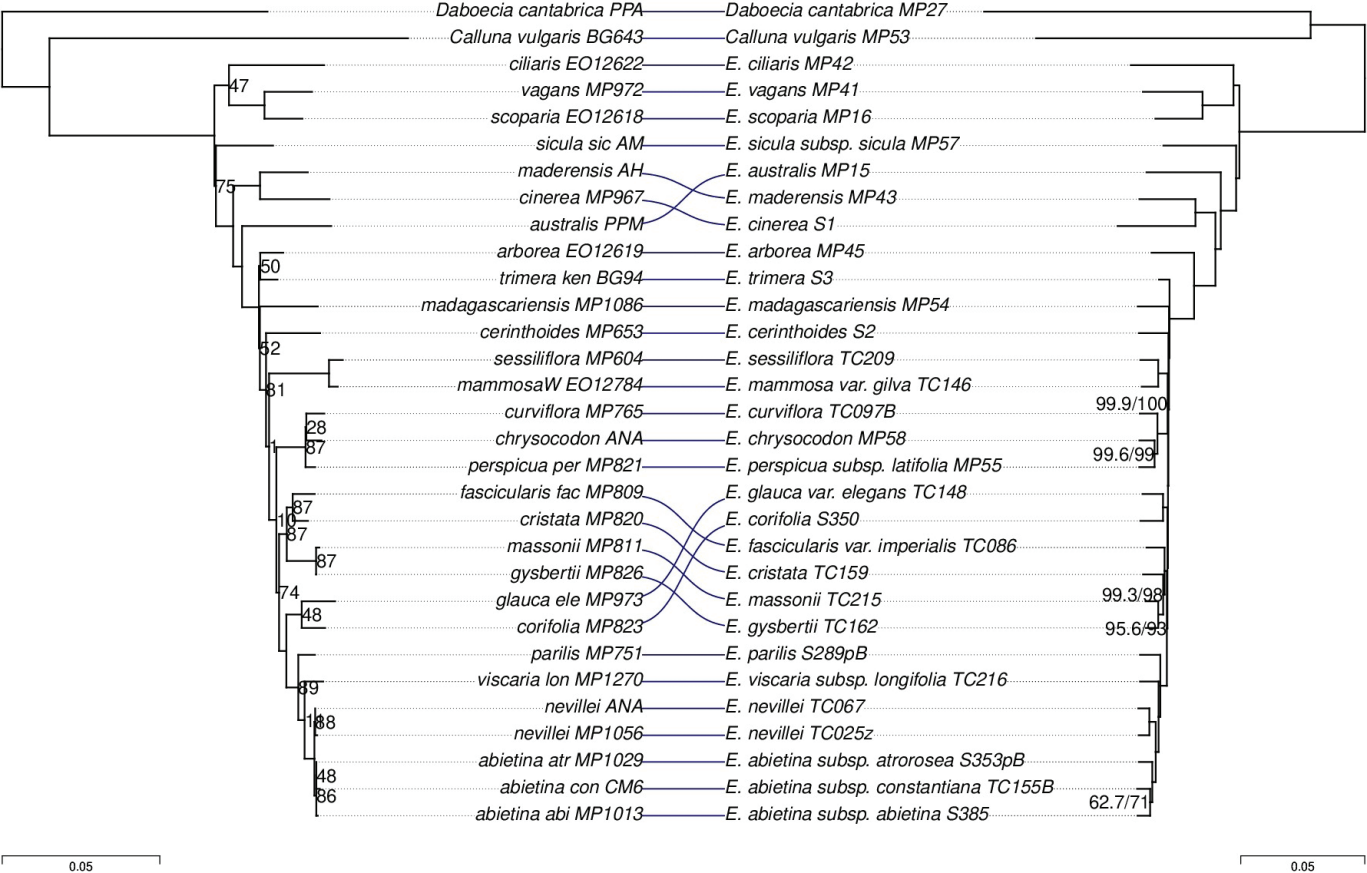
Tanglegram comparing the phylogenies inferred by Pirie et al. using traditional markers (Left) and by concatenation using the Erica285 superset (Right). For the Pirie tree, branch lengths are in substitutions per site and node labels show bootstrap percentage. For the concatenation tree, node values indicate SH-alrt/UFBoot when either value was less than 100%.

**Figure 8.**
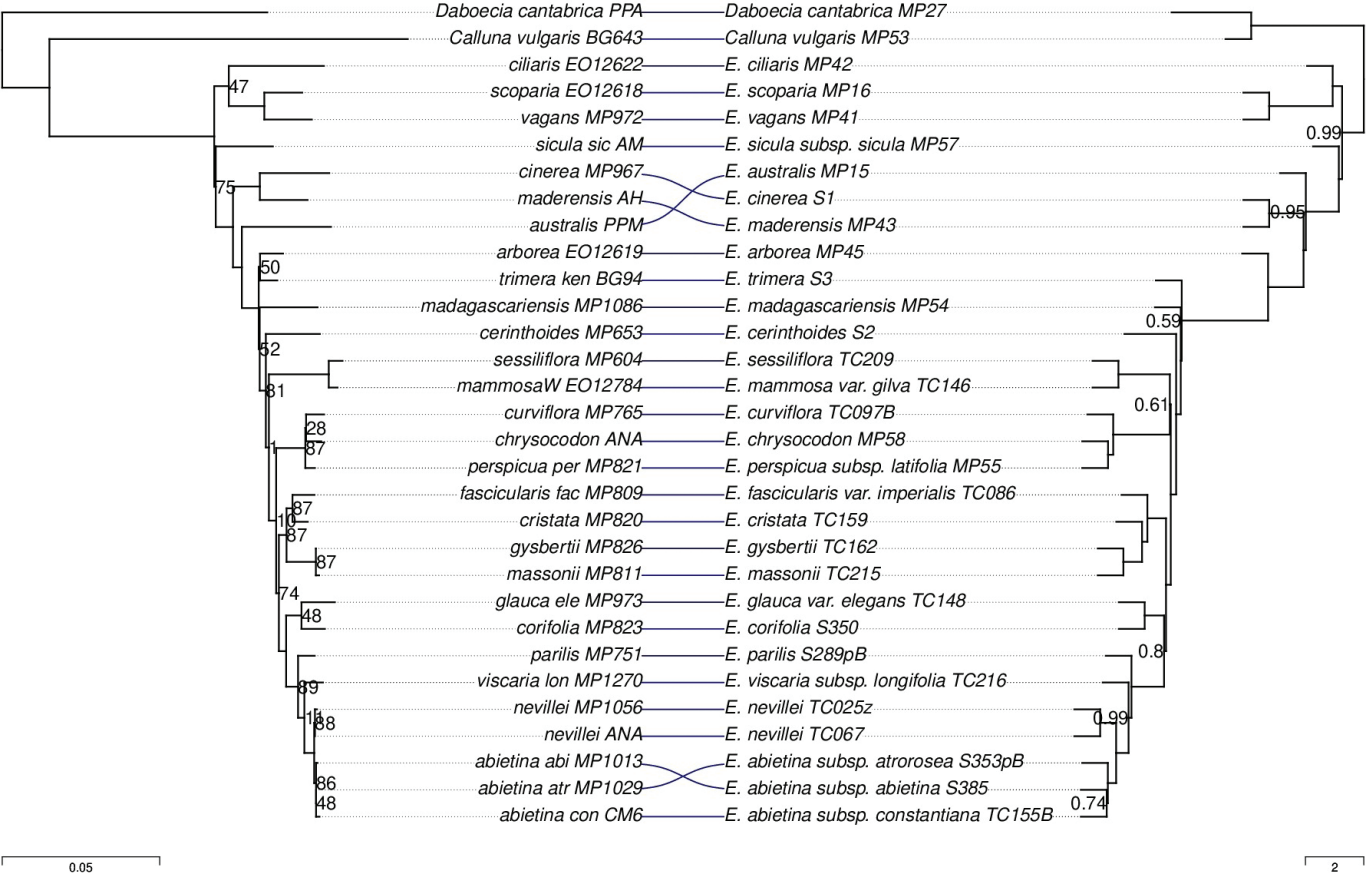
Tanglegram comparing the phylogenies inferred by Pirie et al. using traditional markers (Left) and by ASTRAL using the Erica285 superset (Right). For the Pirie tree, branch lengths are in substitutions per site and node labels show bootstrap percentage. For the ASTRAL tree, node values indicate local posterior probability values below 1.

There were also some discrepancies between the “traditional” marker-based phylogeny of Pirie et al. (2024, hereafter “Pirie tree”) and the phylogenies inferred here (Figs 7, 8). Regarding the “VMC clade”, the Pirie tree agreed with the ASTRAL tree topology (M,(C,V)). On the other hand, both concatenation and ASTRAL inferred a different placement of *E.australis* than the Pirie tree, a conflict that was strongly supported according to branch support values. There were also some much weaker conflicts. For example, the Pirie tree grouped *E.trimera* with *E.arborea* with low support (bootstrap = 50%), whereas the phylogenies inferred here confidently placed *E.arborea* outside the clade of African and Madagascan species.

In summary, there were some topological conflicts between the Pirie tree and the newly inferred trees, as well as between the trees inferred by different methods using the new targets, but only one of the conflicting relationships (the placement of *E.australis*) was strongly supported. Overall, the relationships inferred using the new targets were mostly concordant with prior expectations based on previous work and also produced much more strongly supported topologies, with limited conflict within the “VMC clade” localised at a single node surrounded by very short branches.

### ﻿Phylogenetic informativeness

#### ﻿Parsimony informative sites

Table 3 shows that the supercontig alignments from CDS-derived targets had a significantly smaller total number of PI sites than did the genome-derived alignments for the MarkerMiner and NewTargets sets, but significantly more for the Refinement sets (MarkerMiner, mean difference = -130 sites; NewTargets, mean difference = -223 sites; Refinement, mean difference = 180 sites). In contrast, the proportion of PI sites was slightly greater in CDS-derived alignments for all methods, though the mean difference never exceeded 1%. However, R^2^ values were low for all models, indicating that, overall, PI did not depend strongly on target source.

**Table 3.** Results of the fixed effects models of parsimony-informative (PI) sites (number and proportion) as a function of target source for supercontig alignments, using the Erica285 set. More PI sites were recovered by *Erica* genome-derived targets identified using NewTargets and MarkerMiner, whereas fewer were recovered using the Refinement method. In contrast, the proportion of PI sites was slightly greater in *Rhododendron* CDS-derived targets for all methods, though the mean difference never exceeded 1%. Numbers in brackets are standard errors.

	MarkerMiner		NewTargets		Refinement	
	Number	Prop. (%)	Number	Prop. (%)	Number	Prop. (%)
(Intercept)	717.3***	9.44***	720.8***	8.80***	374.2***	9.11
	(44.3)	(0.217)	(41.2)	(0.172)	(25.6)	(0.143)
Source = *Rhododendron* CDS	-130.2*	0.491	-223.5***	0.598**	180.5**	0.802
	(72.3)	(0.354)	(71.4)	(0.299)	(70.2)	(0.393)
Observations	109	109	78	78	98	98
R2	0.029	0.018	0.114	0.050	0.064	0.042
Adjusted R^2^	0.020	0.008	0.103	0.038	0.055	0.032

Signif. codes: *** = 0.01, ** = 0.05, * = 0.10.

#### ﻿Quartet internode resolution probability

**Figure 9.**
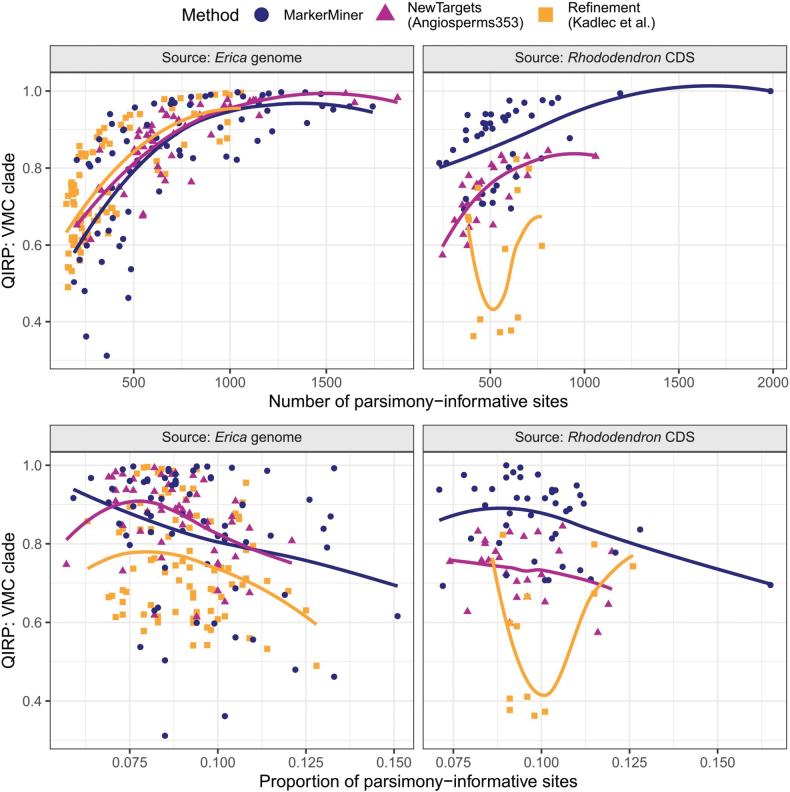
Quartet Internode Resolution Probability (QIRP) at the crown of the “VMC clade” in relation to number (top) and proportion (bottom) of parsimony-informative sites, and target source and method. Lines show loess model fits with span = 1.

Overall, informativeness as measured by QIRP was relatively high (mean = 0.80 ± 0.15 SD), indicating that the target set was informative for young, short internodes. The proportion of PI sites showed no relationship with QIRP, whereas the total number of PI sites showed a strong positive correlation with QIRP (Fig. 9). While the shape of the relationship between QIRP and total PI sites was the same for all methods for the genome-derived alignments, it differed between methods for the CDS-derived alignments (Fig. 9). Specifically, genome-derived alignments showed an asymptotic trend for all three methods, with QIRP increasing until ca. 1,000 PI sites, at which point most alignments had QIRP > 0.9. CDS-derived alignments showed a mixture of trends. The MarkerMiner alignments fell into two distinct groups, one with higher QIRP regardless of PI, though both groups showed a positive trend. The NewTargets alignments had lower QIRP than their genome-derived counterparts, matching the low-QIRP group of MarkerMiner alignments in trend and absolute values. The Refinement alignments showed no clear trend, though they generally had much lower QIRP than the other methods. The smaller range of PI sites for the CDS-derived alignments is important to note, as most had fewer than 1,000 PI sites, the point at which genome-derived alignments reached consistent QIRP highs.

**Figure 10.**
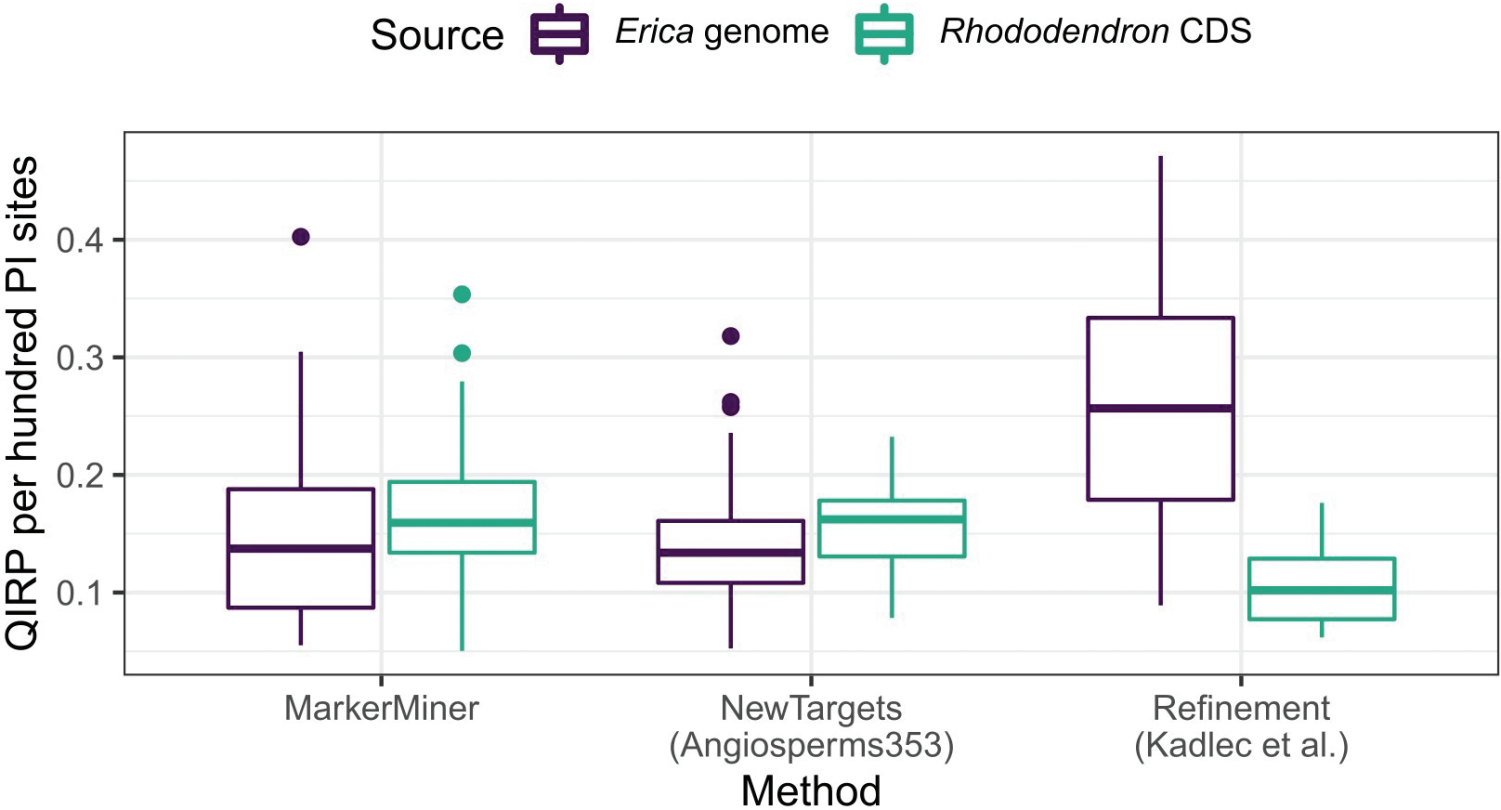
QIRP per hundred PI sites in relation to target source and method.

For a given number of PI sites, QIRP values of genome-derived alignments were much higher than those of CDS-derived alignments for the Refinement set (linear model: F(1,96) = 27.0, R^2^ = 0.21, p < 0.001), but not for the other sets (NewTargets: F(1,76) = 2.82, R^2^ = 0.023, p = 0.097; MarkerMiner: F(1,107) = 2.93, R^2^ = 0.018, p = 0.090; Fig. 10). This revealed that, despite their shorter lengths, the Refinement targets produced relatively more informative alignments per nucleotide base pair.

#### ﻿QIRP and introns

**Figure 11.**
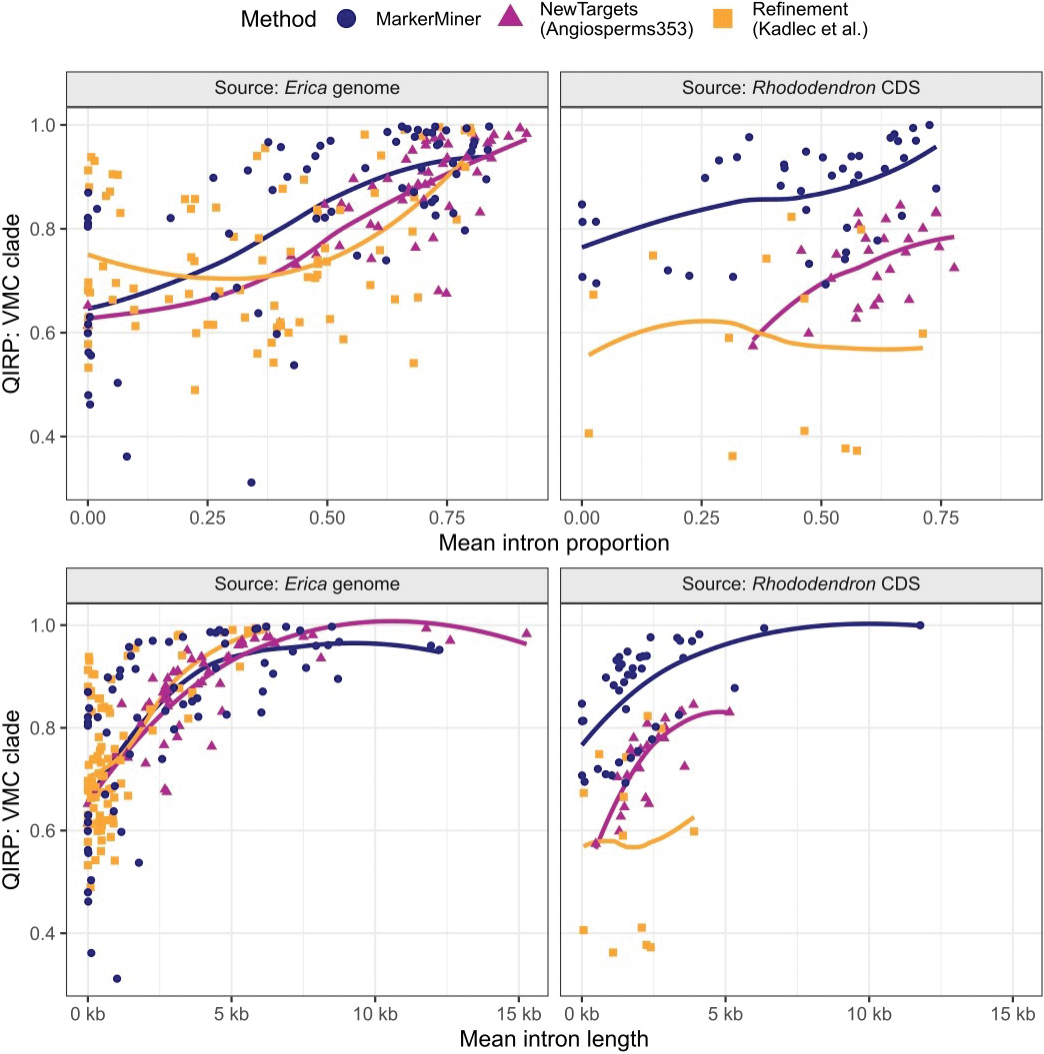
QIRP at the crown of the “VMC clade” in relation to the proportion of intronic sequence, target source and method. Lines show loess model fits with span = 1.

Regardless of target source, the proportion of intron sequence had a strong and significant positive relationship to QIRP (Fig. 11) for the NewTargets alignments (best-fit linear model: QIRP ~ intron prop. + source, F(2,75) = 65.2, R^2^ = 0.63, p < 0.001) and a weaker but still significant relationship for the Refinement alignments (best-fit linear model: QIRP ~ intron prop. + source, F(2,95) = 11.4, R^2^ = 0.18, p < 0.001). The same positive relationship applied to the MarkerMiner alignments except that its slope varied with source (best-fit linear model: QIRP ~ intron prop. * source, F(3,105) = 24.9, R^2^ = 0.40, p < 0.001), though the slope difference was only near-significant (difference = -0.17 ± 0.097 SD, t = -1.77, p = 0.079).

## ﻿Discussion

We developed and tested a new target set for *Erica* phylogenomics using a variety of methods. Overall, we were able to implement effective measures that kept the rate of paralogy and missingness in the resulting target capture data to very low levels. Post-assembly refinement of the target set only slightly reduced the number of targets from 303 to 285, suggesting that the target design approaches effectively identified most undesirable loci. Furthermore, good target recovery in the three non-*Erica* samples tested (*Rhododendronrex*, *Callunavulgaris*, and *Daboeciacantabrica*) suggests that the targets could also be applied to these genera, and perhaps even to more distant relatives (i.e., in Ericaceae beyond the Ericoideae). In the supplementary data we also provide a version of the target set including only the *Rhododendron*-derived, intron-free targets, which users may prefer as a more conservative option when working with *Rhododendron* or other Ericoideae.

Our results demonstrate that the new target set has excellent phylogenetic informativeness. Notably, one of the major reasons for this was the inclusion of intronic sequences in ca. 70% of the targets used for bait design. Although this approach has rarely been attempted (de Sousa et al. 2014; Folk et al. 2015), we observed high intron capture efficiency even for Cape *Erica* species, despite the target source being a European *Erica* more than 40 million years diverged (Pirie et al. 2016). Targeting introns improved their downstream assembly and contiguity, as targets including introns recovered a larger proportion of intronic sequence relative to target length (Fig. 5, Table 2). Finally, the proportion of intronic sequence correlated positively with phylogenetic informativeness (Fig. 11). These results should encourage researchers working in phylogenomics to include introns in their targets, where possible, in order to improve the phylogenetic informativeness of their data.

Comparing target design methods (MarkerMiner, NewTargets, and Refinement) revealed certain differences in phylogenetic informativeness that are not easy to explain. While the Refinement method (i.e., designing targets from supercontigs assembled from a previous target capture experiment) produced loci with the highest QIRP per number of PI sites on average (Fig. 10), the thirteen targets retained in their original form from the Kadlec et al. (2017) set included five of the least informative loci (QIRP at or below 0.4; Figs 9, 11). In other words, the targets designed by Kadlec et al. (2017) went from being among the least to among the most informative loci when switching from *R.scopulorum* transcripts to *E.grandiflora* supercontigs. The poor performance of the original targets might be due to the design choices made by Kadlec et al. (2017), who ranked potential targets by total length, including mean intron length (which was inferred using WGS data of *Ericaplukenetii*; Le Maitre et al. 2019; data not available), but which could nevertheless deliver long but invariable coding sequences. Another unusual result was the relatively low QIRP of Angiosperms353 targets derived from *Rhododendron* transcripts compared to their MarkerMiner counterparts, which was not explained by differences in the number of parsimony-informative sites or intron content (Figs 9, 11). As we measured QIRP at a young node in the tree, this may imply that the variation captured by Angiosperms353 targets reflects deeper phylogenetic splits than that captured by MarkerMiner genes. These comparisons should not, however, be taken as conclusive, as targets were chosen to maximise overall informativeness rather than for the specific purpose of comparison between target sets.

Looking beyond our specific target set, we expect that the target design methods presented here are generally applicable to any plant group. These include (i) using the NewTargets method of McLay et al. (2021) for Angiosperms353 target discovery, (ii) using assembled targets from a closer relative to iteratively refine an earlier target set (Kadlec et al. 2017), (iii) filtering candidate targets based on predictions of copy number derived from WGS reads, and (iv) using assembled genomic contigs to include introns in our targets. Most notably, we found that including introns improved target recovery, that intron content was positively correlated with phylogenetic informativeness, and that WGS reads and draft genomes could be used to good effect to design a target set with low paralogy and missingness. To aid others in implementing several of these approaches, we developed and made freely available an open-source toolkit, TargetVet.

## ﻿Additional information

### Author ORCIDs

Seth D. Musker https://orcid.org/0000-0002-1456-1373

Nicolai M. Nürk https://orcid.org/0000-0002-0471-644X

Michael D. Pirie https://orcid.org/0000-0003-0403-4470

### Data availability

Raw reads are deposited in SRA under BioProject PRJNA1164706. Other associated data are deposited on FigShare at https://doi.org/10.25375/uct.27134208.

## Supplementary materials

Supplementary material 1 Supporting figures and tables Data type pdf Explanation note **figure S1**: Graphical illustration of the functionality of TargetVet. **figure S2**: Venn diagram showing the number of genes initially identified by MarkerMiner for each of the three *Rhododendron* transcriptomes. **figure S3**: Heatmap showing the number of paralogs (i.e., number of gene copies) identified by HybPiper’s length-based method, in which a targets is flagged for a given sample if its secondlongest assembled contig is more than 70% the length of its longest assembled contig. Targets and samples are arranged by mean number of copies. **figure S4**: Paralogy (*P*) estimated using the actual target sequences *versus* using their CDS versions. The solid line shows the linear regression line while the dashed line shows the 1:1 line. Points colours indicate missingness (*M*). **figure S5**: Tanglegram comparing the phylogenies inferred by concatenation (IQ-TREE; Left) and by ASTRAL (Right) using the full Erica303 target superset. For the concatenation tree, branch lengths are in substitutions per site and node labels are SH-alrt/UFBoot percentages. For the ASTRAL tree, branch lengths represent coalescent units (except for terminal branches which are are arbitrarily set to 1 as they are not estimated by ASTRAL) and node labels show posterior probability support. Nodes with full support are unlabelled. The trees are fully bifurcating and are rooted along the branch between the *Erica* and non-*Erica* samples arbitrarily for display purposes. **table S1**: Results of the fixed effects models of supercontig length as a function of target source showing that longer supercontigs were recovered by *Erica* genome-derived targets identified using NewTargets and MarkerMiner, whereas longer supercontigs were recovered by *Rhododendron* CDS-derived targets identified using the Refinement method. *R*^2^ indicates the fit of the full model, while Within *R*^2^ indicates the fit when fixed effects are ignored. Numbers in brackets are standard errors. **table S2**: Voucher information. Unless otherwise noted, collections were made by the author. Specimens have been deposited at NBG. https://binary.pensoft.net/file/1221484This dataset is made available under the Open Database License (http://opendatacommons.org/licenses/odbl/1.0/). The Open Database License (ODbL) is a license agreement intended to allow users to freely share, modify, and use this Dataset while maintaining this same freedom for others, provided that the original source and author(s) are credited.Seth D. Musker, Nicolai M. Nürk, Michael D. Pirie

## References

[B1] Altschul SF Madden TL Schäffer AA Zhang J Zhang Z Miller W Lipman DJ (1997) Gapped BLAST and PSI-BLAST: A new generation of protein database search programs. Nucleic Acids Research 25(17): 3389–3402. 10.1093/nar/25.17.3389 9254694 PMC146917

[B2] Avise JC Arnold J Ball RM Bermingham E Lamb T Neigel JE Reeb CA Saunders NC (1987) Intraspecific phylogeography: The Mitochondrial DNA Bridge Between Population Genetics and Systematics. Annual Review of Ecology and Systematics 18(1): 489–522. 10.1146/annurev.es.18.110187.002421

[B3] Bagley JC Uribe-Convers S Carlsen MM Muchhala N (2020) Utility of targeted sequence capture for phylogenomics in rapid, recent angiosperm radiations: Neotropical *Burmeistera* bellflowers as a case study. Molecular Phylogenetics and Evolution 152: 106769. 10.1016/j.ympev.2020.106769 32081762

[B4] Baker WJ Bailey P Barber V Barker A Bellot S Bishop D Botigué LR Brewer G Carruthers T Clarkson JJ Cook J Cowan RS Dodsworth S Epitawalage N Françoso E Gallego B Johnson MG Kim JT Leempoel K Maurin O Mcginnie C Pokorny L Roy S Stone M Toledo E Wickett NJ Zuntini AR Eiserhardt WL Kersey PJ Leitch IJ Forest F (2021) A comprehensive phylogenomic platform for exploring the angiosperm tree of life. Systematic Biology 71(2): 301–319. 10.1093/sysbio/syab035 PMC8830076 33983440

[B5] Bankevich A Nurk S Antipov D Gurevich AA Dvorkin M Kulikov AS Lesin VM Nikolenko SI Pham S Prjibelski AD Pyshkin AV Sirotkin AV Vyahhi N Tesler G Alekseyev MA Pevzner PA (2012) SPAdes: A new genome assembly algorithm and its applications to single-cell sequencing. Journal of Computational Biology 19(5): 455–477. 10.1089/cmb.2012.0021 22506599 PMC3342519

[B6] Barnett DW Garrison EK Quinlan AR Strömberg MP Marth GT (2011) BamTools: A C++ API and toolkit for analyzing and managing BAM files. Bioinformatics (Oxford, England) 27(12): 1691–1692. 10.1093/bioinformatics/btr174 21493652 PMC3106182

[B7] Bergé L (2018) Efficient estimation of maximum likelihood models with multiple fixed-effects: the R package FENmlm. Technical report, Department of Economics at the University of Luxembourg.

[B8] Borowiec ML (2016) AMAS: A fast tool for alignment manipulation and computing of summary statistics. Peerj 2016(1). 10.7717/peerj.1660 PMC4734057 26835189

[B9] Chamala S García N Godden GT Krishnakumar V Jordon-Thaden IE Smet RD Barbazuk WB Soltis DE Soltis PS (2015) MarkerMiner 1.0: A new application for phylogenetic marker development using angiosperm transcriptomes. Applications in Plant Sciences 3(4): 1400115. 10.3732/apps.1400115 PMC4406834 25909041

[B10] Charif D Lobry J (2007) SeqinR 1.0-2: a contributed package to the R project for statistical computing devoted to biological sequences retrieval and analysis. In: U Bastolla, M Porto, H Roman, Vendruscolo M (Eds) Structural approaches to sequence evolution: Molecules, networks, populations, Springer Verlag, New York, 207–232. 10.1007/978-3-540-35306-5_10

[B11] Chen S Zhou Y Chen Y Gu J (2018) fastp: An ultra-fast all-in-one FASTQ preprocessor. Bioinformatics (Oxford, England) 34(17): i884–i890. 10.1093/bioinformatics/bty560 PMC6129281 30423086

[B12] Costa L Marques A Buddenhagen C Thomas WW Huettel B Schubert V Dodsworth S Houben A Souza G Pedrosa-Harand A (2021) Aiming off the target: Recycling target capture sequencing reads for investigating repetitive DNA. Annals of Botany 128(7): 835–848. 10.1093/aob/mcab063 34050647 PMC8577205

[B13] Danecek P Bonfield JK Liddle J Marshall J Ohan V Pollard MO Whitwham A Keane T McCarthy SA Davies RM Li H (2021) Twelve years of SAMtools and BCFtools. GigaScience 10(2): giab008. 10.1093/gigascience/giab008 PMC7931819 33590861

[B14] De Smet R Adams KL Vandepoele K Montagu MCEV Maere S de Peer YV (2013) Convergent gene loss following gene and genome duplications creates single-copy families in flowering plants. Proceedings of the National Academy of Sciences of the United States of America 110(8): 2898–2903. 10.1073/pnas.1300127110 23382190 PMC3581894

[B15] de Sousa F Bertrand YJK Nylinder S Oxelman B Eriksson JS Pfeil BE (2014) Phylogenetic Properties of 50 Nuclear Loci in *Medicago* (Leguminosae) Generated Using Multiplexed Sequence Capture and Next-Generation Sequencing. PLOS ONE 9(10): e109704. 10.1371/journal.pone.0109704 PMC4201463 25329401

[B16] Degnan JH Rosenberg NA (2006) Discordance of species trees with their most likely gene trees. PLOS Genetics 2(5): e68. 10.1371/journal.pgen.0020068 PMC1464820 16733550

[B17] Degnan JH Rosenberg NA (2009) Gene tree discordance, phylogenetic inference and the multispecies coalescent. Trends in Ecology & Evolution 24(6): 332–340. 10.1016/j.tree.2009.01.009 19307040

[B18] Dornburg A Fisk JN Tamagnan J Townsend JP (2016) PhyInformR: Phylogenetic experimental design and phylogenomic data exploration in R. BMC Evolutionary Biology 16(1): 262. 10.1186/s12862-016-0837-3 27905871 PMC5134231

[B19] Elliott AC Bester SP Klopper RR Nelson EC Pirie MD (2024) Curating an online checklist for *Erica* L. (Ericaceae): Contributing to and supporting global conservation through the World Flora Online. PhytoKeys 243: 121–135. 10.3897/phytokeys.243.121555 38947554 PMC11214009

[B20] Ewels P Magnusson M Lundin S Käller M (2016) MultiQC: Summarize analysis results for multiple tools and samples in a single report. Bioinformatics (Oxford, England) 32(19): 3047–3048. 10.1093/bioinformatics/btw354 27312411 PMC5039924

[B21] Fernández R Gabaldon T Dessimoz C (2020) Orthology: Definitions, Prediction, and Impact on Species Phylogeny Inference. In: Scornavacca C, Delsuc F, Galtier N (Eds) Phylogenetics in the Genomic Era, No commercial publisher, Authors open access book, 2.4:1–2.4:14. https://hal.science/hal-02535414

[B22] Fitch WM (1970) Distinguishing homologous from analogous proteins. Systematic Zoology 19(2): 99–113. 10.2307/2412448 5449325

[B23] Folk RA Mandel JR Freudenstein JV (2015) A protocol for targeted enrichment of intron-containing sequence markers for recent radiations: A phylogenomic example from *Heuchera* (Saxifragaceae). Applications in Plant Sciences 3(8): 1500039. 10.3732/apps.1500039 PMC4542943 26312196

[B24] Gardner EM (2021) HerbChomper: A bioinformatic tool for trimming poorly-aligned ends from DNA sequences. https://github.com/artocarpus/HerbChomper [Accessed 3 December 2021]

[B25] Gardner EM Johnson MG Pereira JT Puad ASA Arifiani D Sahromi Wickett NJ Zerega NJC (2021) Paralogs and off-target sequences improve phylogenetic resolution in a densely sampled study of the breadfruit genus (*Artocarpus*, Moraceae). Systematic Biology 70(3): 558–575. 10.1093/sysbio/syaa073 PMC8048387 32970819

[B26] Gnirke A Melnikov A Maguire J Rogov P LeProust EM Brockman W Fennell T Giannoukos G Fisher S Russ C Gabriel S Jaffe DB Lander ES Nusbaum C (2009) Solution hybrid selection with ultra-long oligonucleotides for massively parallel targeted sequencing. Nature Biotechnology 27(2): 182–189. 10.1038/nbt.1523 PMC2663421 19182786

[B27] Guindon S Dufayard JF Lefort V Anisimova M Hordijk W Gascuel O (2010) New algorithms and methods to estimate maximum-likelihood phylogenies: Assessing the performance of PhyML 3.0. Systematic Biology 59(3): 307–321. 10.1093/sysbio/syq010 20525638

[B28] Hoang DT Chernomor O Von Haeseler A Minh BQ Vinh LS (2018) UFBoot2: Improving the ultrafast bootstrap approximation. Molecular Biology and Evolution 35(2): 518–522. 10.1093/molbev/msx281 29077904 PMC5850222

[B29] Jackman SD Vandervalk BP Mohamadi H Chu J Yeo S Hammond SA Jahesh G Khan H Coombe L Warren RL Birol I (2017) ABySS 2.0: Resource-efficient assembly of large genomes using a bloom filter. Genome Research 27(5): 768–777. 10.1101/gr.214346.116 28232478 PMC5411771

[B30] Johnson MG Gardner EM Liu Y Medina R Goffinet B Shaw AJ Zerega NJ Wickett NJ (2016) HybPiper: Extracting coding sequence and introns for phylogenetics from high-throughput sequencing reads using target enrichment. Applications in Plant Sciences 4(7): 1600016. 10.3732/apps.1600016 PMC4948903 27437175

[B31] Johnson MG Pokorny L Dodsworth S Botigué LR Cowan RS Devault A Eiserhardt WL Epitawalage N Forest F Kim JT Leebens-Mack JH Leitch IJ Maurin O Soltis DE Soltis PS Wong GKS Baker WJ Wickett NJ (2019) A universal probe set for targeted sequencing of 353 Nuclear genes from any flowering plant designed using k-medoids clustering. Systematic Biology 68(4): 594–606. 10.1093/sysbio/syy086 30535394 PMC6568016

[B32] Jones KE Fér T Schmickl RE Dikow RB Funk VA Herrando-Moraira S Johnston PR Kilian N Siniscalchi CM Susanna A Slovák M Thapa R Watson LE Mandel JR (2019) An empirical assessment of a single family-wide hybrid capture locus set at multiple evolutionary timescales in Asteraceae. Applications in Plant Sciences 7(10): e11295. 10.1002/aps3.11295 PMC6814182 31667023

[B33] Kadlec M Bellstedt DU Le Maitre NC Pirie MD (2017) Targeted NGS for species level phylogenomics: “made to measure” or “one size fits all”? PeerJ 5: e3569. 10.7717/peerj.3569 PMC5530999 28761782

[B34] Kalyaanamoorthy S Minh BQ Wong TK Von Haeseler A Jermiin LS (2017) ModelFinder: Fast model selection for accurate phylogenetic estimates. Nature Methods 14(6): 587–589. 10.1038/nmeth.4285 28481363 PMC5453245

[B35] Karin BR Gamble T Jackman TR (2019) Optimizing phylogenomics with rapidly evolving long exons: Comparison with anchored hybrid enrichment and ultraconserved elements. Molecular Biology and Evolution 37(3): 904–922. 10.1093/molbev/msz263 PMC7038749 31710677

[B36] Karin EL Mirdita M Söding J (2020) MetaEuk—Sensitive, high-throughput gene discovery, and annotation for large-scale eukaryotic metagenomics. Microbiome 8(1): 48. 10.1186/s40168-020-00808-x 32245390 PMC7126354

[B37] Katoh K Standley DM (2013) MAFFT multiple sequence alignment software version 7: Improvements in performance and usability. Molecular Biology and Evolution 30(4): 772–780. 10.1093/molbev/mst010 23329690 PMC3603318

[B38] Lanfear R Calcott B Kainer D Mayer C Stamatakis A (2014) Selecting optimal partitioning schemes for phylogenomic datasets. BMC Evolutionary Biology 14(1): 1–14. 10.1186/1471-2148-14-82 PMC4012149 24742000

[B39] Le Maitre NC Pirie MD Bellstedt DU (2019) An approach to determining anthocyanin synthesis enzyme gene expression in an evolutionary context: An example from *Ericaplukenetii*. Annals of Botany 124(1): 121–130. 10.1093/aob/mcz046 31008513 PMC6676384

[B40] Li H (2013) Aligning sequence reads, clone sequences and assembly contigs with BWA-MEM. Arxiv:1303.3997. http://arxiv.org/abs/1303.3997

[B41] Linder HP (2003) The radiation of the Cape flora, southern Africa. Biological Reviews of the Cambridge Philosophical Society 78(4): 597–638. 10.1017/S1464793103006171 14700393

[B42] Maddison WP (1997) Gene trees in species trees. Systematic Biology 46(3): 523–536. 10.1093/sysbio/46.3.523

[B43] Madeira F Pearce M Tivey ARN Basutkar P Lee J Edbali O Madhusoodanan N Kolesnikov A Lopez R (2022) Search and sequence analysis tools services from EMBL-EBI in 2022. Nucleic Acids Research 50(W1): W276–W279. 10.1093/nar/gkac240 PMC9252731 35412617

[B44] Manning J Goldblatt P (2012) Plants of The Greater Cape Floristic Region 1: The Core Cape Flora, volume 29. South African National Biodiversity Institute, Pretoria. 10.1017/CBO9781107415324.004

[B45] Matasci N Hung LH Yan Z Carpenter EJ Wickett NJ Mirarab S Nguyen N Warnow T Ayyampalayam S Barker M Burleigh JG Gitzendanner MA Wafula E Der JP dePamphilis CW Roure B Philippe H Ruhfel BR Miles NW Graham SW Mathews S Surek B Melkonian M Soltis DE Soltis PS Rothfels C Pokorny L Shaw JA DeGironimo L Stevenson DW Villarreal JC Chen T Kutchan TM Rolf M Baucom RS Deyholos MK Samudrala R Tian Z Wu X Sun X Zhang Y Wang J Leebens-Mack J Wong GKS (2014) Data access for the 1,000 Plants (1KP) project. GigaScience 3(1): 17. 10.1186/2047-217X-3-17 25625010 PMC4306014

[B46] Maurin O Anest A Bellot S Biffin E Brewer G Charles-Dominique T Cowan RS Dodsworth S Epitawalage N Gallego B Giaretta A Goldenberg R Gonçalves DJP Graham S Hoch P Mazine F Low YW McGinnie C Michelangeli FA Morris S Penneys DS Pérez Escobar OA Pillon Y Pokorny L Shimizu G Staggemeier VG Thornhill AH Tomlinson KW Turner IM Vasconcelos T Wilson PG Zuntini AR Baker WJ Forest F Lucas E (2021) A nuclear phylogenomic study of the angiosperm order Myrtales, exploring the potential and limitations of the universal Angiosperms353 probe set. American Journal of Botany 108(7): 1087–1111. 10.1002/ajb2.1699 34297852

[B47] McLay TG Birch JL Gunn BF Ning W Tate JA Nauheimer L Joyce EM Simpson L Schmidt-Lebuhn AN Baker WJ Forest F Jackson CJ (2021) New targets acquired: Improving locus recovery from the Angiosperms353 probe set. Applications in Plant Sciences 9(7): aps3.11420. 10.1002/aps3.11420 PMC8312740 34336399

[B48] Minh BQ Schmidt HA Chernomor O Schrempf D Woodhams MD Von Haeseler A Lanfear R Teeling E (2020) IQ-TREE 2: New models and efficient methods for phylogenetic inference in the genomic era. Molecular Biology and Evolution 37(5): 1530–1534. 10.1093/molbev/msaa015 32011700 PMC7182206

[B49] Mistry J Finn RD Eddy SR Bateman A Punta M (2013) Challenges in homology search: HMMER3 and convergent evolution of coiled-coil regions. Nucleic Acids Research 41(12): e121–e121. 10.1093/nar/gkt263 PMC3695513 23598997

[B50] Morgan M Pagès H Obenchain V Hayden N (2021) Rsamtools: Binary alignment (BAM), FASTA, variant call (BCF), and tabix file import. https://bioconductor.org/packages/Rsamtools

[B51] Mugrabi De Kuppler AL (2013) Phylogenetics, flow-cytometry and pollen storage in *Erica* L. (Ericaceae). PhD Thesis, 76 pp.

[B52] Musker SD Pirie MD Nürk NM (2024) Pollinator shifts despite hybridisation in the Cape’s hyperdiverse heathers (*Erica*, Ericaceae). Molecular Ecology 33(18): e17505. 10.1111/mec.17505 39188071

[B53] Nelson EC Oliver EGH (2005) Chromosome numbers in *Erica* - an updated checklist. Yearbook of the Heather Society 2005 (volume 53). Heather Society, Ipswich, 57–58. https://www.cabidigitallibrary.org/doi/full/10.5555/20053124100

[B54] Oliver EGH Forshaw N Oliver IM Volk F Schumann AWS Dorr LJ Hoekstra RD Musker SD Nürk NM Pirie MD Rebelo AG (2024) Genus *Erica*: An identification aid version 4.00. PhytoKeys 241: 143–154. 10.3897/phytokeys.241.117604 38699680 PMC11063621

[B55] Paradis E (2013) Molecular dating of phylogenies by likelihood methods: A comparison of models and a new information criterion. Molecular Phylogenetics and Evolution 67(2): 436–444. 10.1016/j.ympev.2013.02.008 23454091

[B56] Paradis E Schliep K (2019) ape 5.0: An environment for modern phylogenetics and evolutionary analyses in R. Bioinformatics (Oxford, England) 35(3): 526–528. 10.1093/bioinformatics/bty633 30016406

[B57] Pirie MD Oliver EGH Bellstedt DU (2011) A densely sampled ITS phylogeny of the Cape flagship genus *Erica* L. suggests numerous shifts in floral macro-morphology. Molecular Phylogenetics and Evolution 61(2): 593–601. 10.1016/j.ympev.2011.06.007 21722743

[B58] Pirie MD Oliver EGH Kuppler AMD Gehrke B Maitre NCL Kandziora M Bellstedt DU (2016) The biodiversity hotspot as evolutionary hot-bed: Spectacular radiation of *Erica* in the Cape Floristic Region. BMC Evolutionary Biology 16(1): 190. 10.1186/s12862-016-0764-3 27639849 PMC5027107

[B59] Pirie MD Oliver EG Gehrke B Heringer L De Kuppler AM Le Maitre NC Bellstedt DU (2017) Underestimated regional species diversity in the Cape Floristic Region revealed by phylogenetic analysis of the *Ericaabietina*/*E.viscaria* clade (Ericaceae). Botanical Journal of the Linnean Society 184(2): 185–203. 10.1093/botlinnean/box021

[B60] Pirie MD Kandziora M Nürk NM Le Maitre NC Mugrabi De Kuppler A Gehrke B Oliver EG Bellstedt DU (2019) Leaps and bounds: geographical and ecological distance constrained the colonisation of the Afrotemperate by *Erica*. BMC Evolutionary Biology 19(1): 222 . 10.1186/s12862-019-1545-6 31805850 PMC6896773

[B61] Pirie MD Bellstedt DU Bouman RW Fagúndez J Gehrke B Kandziora M Le Maitre NC Musker SD Newman E Nürk NM Oliver EGH Pipins S van der Niet T Forest F (2024) Spatial decoupling of taxon richness, phylogenetic diversity and threat status in the megagenus *Erica* (Ericaceae). PhytoKeys 244: 127–150. 10.3897/phytokeys.244.124565 39027483 PMC11255470

[B62] R Core Team (2021) R: A Language and Environment for Statistical Computing. R Foundation for Statistical Computing, Vienna. https://www.R-project.org/

[B63] Revell LJ (2011) phytools: An R package for phylogenetic comparative biology (and other things). Methods in Ecology and Evolution 3(2): 217–223. 10.1111/j.2041-210X.2011.00169.x

[B64] Sedlazeck FJ Rescheneder P Von Haeseler A (2013) NextGenMap: Fast and accurate read mapping in highly polymorphic genomes. Bioinformatics (Oxford, England) 29(21): 2790–2791. 10.1093/bioinformatics/btt468 23975764

[B65] Shah N Nute MG Warnow T Pop M (2019) Misunderstood parameter of NCBI BLAST impacts the correctness of bioinformatics workflows. Bioinformatics (Oxford, England) 35(9): 1613–1614. 10.1093/bioinformatics/bty833 30247621

[B66] Simão FA Waterhouse RM Ioannidis P Kriventseva EV Zdobnov EM (2015) BUSCO: Assessing genome assembly and annotation completeness with single-copy orthologs. Bioinformatics (Oxford, England) 31(19): 3210–3212. 10.1093/bioinformatics/btv351 26059717

[B67] Simpson JT Wong K Jackman SD Schein JE Jones SJ Birol İ (2009) ABySS: A parallel assembler for short read sequence data. Genome Research 19(6): 1117–1123. 10.1101/gr.089532.108 19251739 PMC2694472

[B68] Slater G Birney E (2005) Automated generation of heuristics for biological sequence comparison. BMC Bioinformatics 6(1): 31. 10.1186/1471-2105-6-31 15713233 PMC553969

[B69] Soltis PS Marchant DB de Peer YV Soltis DE (2015) Polyploidy and genome evolution in plants. Current Opinion in Genetics & Development 35: 119–125. 10.1016/j.gde.2015.11.003 26656231

[B70] Soza VL Lindsley D Waalkes A Ramage E Patwardhan RP Burton JN Adey A Kumar A Qiu R Shendure J Hall B (2019) The *Rhododendron* genome and chromosomal organization provide insight into shared whole-genome duplications across the heath family (Ericaceae). Genome Biology and Evolution 11(12): 3353–3371. 10.1093/gbe/evz245 31702783 PMC6907397

[B71] Stamatakis A (2014) RAxML version 8: A tool for phylogenetic analysis and post-analysis of large phylogenies. Bioinformatics (Oxford, England) 30(9): 1312–1313. 10.1093/bioinformatics/btu033 24451623 PMC3998144

[B72] Steenwyk JL Buida III TJ Li Y Shen XX Rokas A (2020) ClipKIT: A multiple sequence alignment trimming software for accurate phylogenomic inference. PLoS Biology 18(12): e3001007. 10.1371/journal.pbio.3001007 PMC7735675 33264284

[B73] Straub SC Boutte J Fishbein M Livshultz T (2020) Enabling evolutionary studies at multiple scales in Apocynaceae through Hyb-Seq. Applications in Plant Sciences 8(11): e11400. 10.1002/aps3.11400 PMC7705337 33304663

[B74] Tan G Muffato M Ledergerber C Herrero J Goldman N Gil M Dessimoz C (2015) Current methods for automated filtering of multiple sequence alignments frequently worsen single-gene phylogenetic inference. Systematic Biology 64(5): 778–791. 10.1093/sysbio/syv033 26031838 PMC4538881

[B75] Tank DC Eastman JM Pennell MW Soltis PS Soltis DE Hinchliff CE Brown JW Sessa EB Harmon LJ (2015) Nested radiations and the pulse of angiosperm diversification: Increased diversification rates often follow whole genome duplications. The New Phytologist 207(2): 454–467. 10.1111/nph.13491 26053261

[B76] Thomas AE Igea J Meudt HM Albach DC Lee WG Tanentzap AJ (2021) Using target sequence capture to improve the phylogenetic resolution of a rapid radiation in New Zealand *Veronica*. American Journal of Botany 108(7): 1289–1306. 10.1002/ajb2.1678 34173225

[B77] Ufimov R Gorospe JM Fér T Kandziora M Salomon L van Loo M Schmickl R (2022) Utilizing paralogues for phylogenetic reconstruction has the potential to increase species tree support and reduce gene tree discordance in target enrichment data. Molecular Ecology Resources 22(8): 3018–3034. 10.1111/1755-0998.13684 35796729

[B78] Wickham H (2016) ggplot2: Elegant Graphics for Data Analysis. Springer, New York. https://ggplot2.tidyverse.org

[B79] Yang FS Nie S Liu H Shi TL Tian XC Zhou SS Bao YT Jia KH Guo JF Zhao W An N Zhang RG Yun QZ Wang XZ Mannapperuma C Porth I El-Kassaby YA Street NR Wang XR Van de Peer Y Mao JF (2020) Chromosome-level genome assembly of a parent species of widely cultivated azaleas. Nature Communications 11(1): 5269. 10.1038/s41467-020-18771-4 PMC7572368 33077749

[B80] Zhang C Mirarab S (2022) Weighting by gene tree uncertainty improves accuracy of quartet-based species trees. Molecular Biology and Evolution 39(12): msac215. 10.1093/molbev/msac215 PMC9750496 36201617

[B81] Zhang L Xu P Cai Y Ma L Li S Li S Xie W Song J Peng L Yan H Zou L Ma Y Zhang C Gao Q Wang J (2017) The draft genome assembly of RhododendrondelavayiFranch.var.delavayi. GigaScience 6(10): gix076. 10.1093/gigascience/gix076 PMC5632301 29020749

[B82] Zhang C Rabiee M Sayyari E Mirarab S (2018) ASTRAL-III: Polynomial time species tree reconstruction from partially resolved gene trees. BMC Bioinformatics 19(S6, Suppl 6): 15–30. 10.1186/s12859-018-2129-y PMC5998893 29745866

[B83] Zhou W Soghigian J Xiang QY (2022) A new pipeline for removing paralogs in target enrichment data. Systematic Biology 71(2): 410–425. 10.1093/sysbio/syab044 34146111 PMC8974407

[B84] Zuntini AR Carruthers T Maurin O Bailey PC Leempoel K Brewer GE Epitawalage N Françoso E Gallego-Paramo B McGinnie C Negrão R Roy SR Simpson L Toledo Romero E Barber VMA Botigué L Clarkson JJ Cowan RS Dodsworth S Johnson MG Kim JT Pokorny L Wickett NJ Antar GM DeBolt L Gutierrez K Hendriks KP Hoewener A Hu AQ Joyce EM Kikuchi IABS Larridon I Larson DA de Lírio EJ Liu JX Malakasi P Przelomska NAS Shah T Viruel J Allnutt TR Ameka GK Andrew RL Appelhans MS Arista M Ariza MJ Arroyo J Arthan W Bachelier JB Bailey CD Barnes HF Barrett MD Barrett RL Bayer RJ Bayly MJ Biffin E Biggs N Birch JL Bogarín D Borosova R Bowles AMC Boyce PC Bramley GLC Briggs M Broadhurst L Brown GK Bruhl JJ Bruneau A Buerki S Burns E Byrne M Cable S Calladine A Callmander MW Cano Á Cantrill DJ Cardinal-McTeague WM Carlsen MM Carruthers AJA de Castro Mateo A Chase MW Chatrou LW Cheek M Chen S Christenhusz MJM Christin P-A Clements MA Coffey SC Conran JG Cornejo X Couvreur TLP Cowie ID Csiba L Darbyshire I Davidse G Davies NMJ Davis AP van Dijk K Downie SR Duretto MF Duvall MR Edwards SL Eggli U Erkens RHJ Escudero M de la Estrella M Fabriani F Fay MF Ferreira PL Ficinski SZ Fowler RM Frisby S Fu L Fulcher T Galbany-Casals M Gardner EM German DA Giaretta A Gibernau M Gillespie LJ González CC Goyder DJ Graham SW Grall A Green L Gunn BF Gutiérrez DG Hackel J Haevermans T Haigh A Hall JC Hall T Harrison MJ Hatt SA Hidalgo O Hodkinson TR Holmes GD Hopkins HCF Jackson CJ James SA Jobson RW Kadereit G Kahandawala IM Kainulainen K Kato M Kellogg EA King GJ Klejevskaja B Klitgaard BB Klopper RR Knapp S Koch MA Leebens-Mack JH Lens F Leon CJ Léveillé-Bourret É Lewis GP Li D-Z Li L Liede-Schumann S Livshultz T Lorence D Lu M Lu-Irving P Luber J Lucas EJ Luján M Lum M Macfarlane TD Magdalena C Mansano VF Masters LE Mayo SJ McColl K McDonnell AJ McDougall AE McLay TGB McPherson H Meneses RI Merckx VSFT Michelangeli FA Mitchell JD Monro AK Moore MJ Mueller TL Mummenhoff K Munzinger J Muriel P Murphy DJ Nargar K Nauheimer L Nge FJ Nyffeler R Orejuela A Ortiz EM Palazzesi L Peixoto AL Pell SK Pellicer J Penneys DS Perez-Escobar OA Persson C Pignal M Pillon Y Pirani JR Plunkett GM Powell RF Prance GT Puglisi C Qin M Rabeler RK Rees PEJ Renner M Roalson EH Rodda M Rogers ZS Rokni S Rutishauser R de Salas MF Schaefer H Schley RJ Schmidt-Lebuhn A Shapcott A Al-Shehbaz I Shepherd KA Simmons MP Simões AO Simões ARG Siros M Smidt EC Smith JF Snow N Soltis DE Soltis PS Soreng RJ Sothers CA Starr JR Stevens PF Straub SCK Struwe L Taylor JM Telford IRH Thornhill AH Tooth I Trias-Blasi A Udovicic F Utteridge TMA Del Valle JC Verboom GA Vonow HP Vorontsova MS de Vos JM Al-Wattar N Waycott M Welker CAD White AJ Wieringa JJ Williamson LT Wilson TC Wong SY Woods LA Woods R Worboys S Xanthos M Yang Y Zhang Y-X Zhou M-Y Zmarzty S Zuloaga FO Antonelli A Bellot S Crayn DM Grace OM Kersey PJ Leitch IJ Sauquet H Smith SA Eiserhardt WL Forest F Baker WJ (2024) Phylogenomics and the rise of the angiosperms. Nature 629(8013): 843–850. 10.1038/s41586-024-07324-0 38658746 PMC11111409

