# Supplementary material for: ﻿Maximising informativeness for target capture-based phylogenomics in Erica (Ericaceae)
Source: PhytoKeys. 2025 Jan 16;251:87–118. doi: 10.3897/phytokeys.251.136373 (PMC11758362; doi:10.3897/phytokeys.251.136373)
Supplement: Supplementary material 1 — Supporting figures and tables [file phytokeys-251-087_article-136373__-s001.pdf]

Supporting Information

for

Worth the fuss? Maximising informativeness for target capture-based phylogenomics in *Erica* (Ericaceae)

1: Supporting Figures

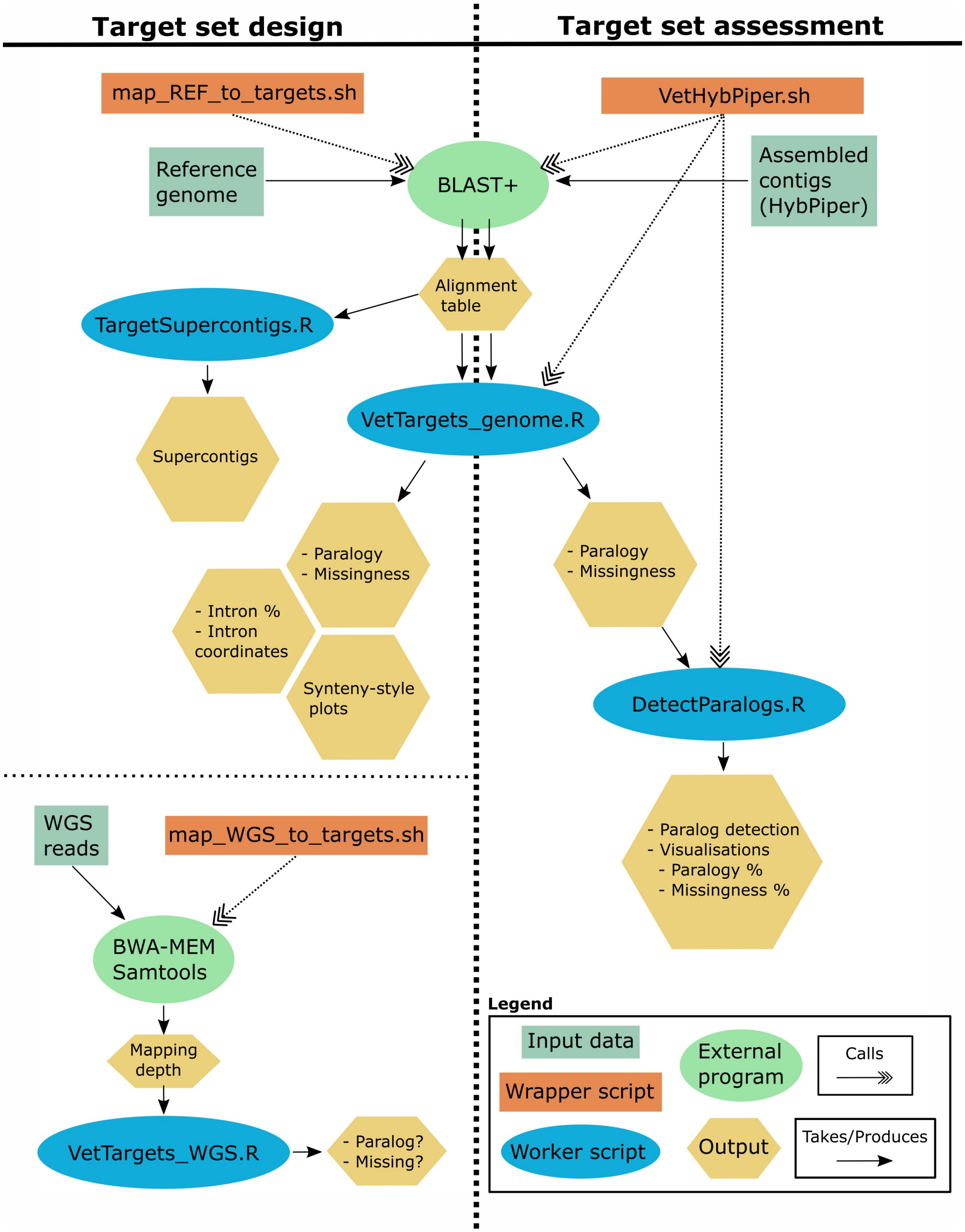

Figure S1 Graphical illustration of the functionality of TargetVet.

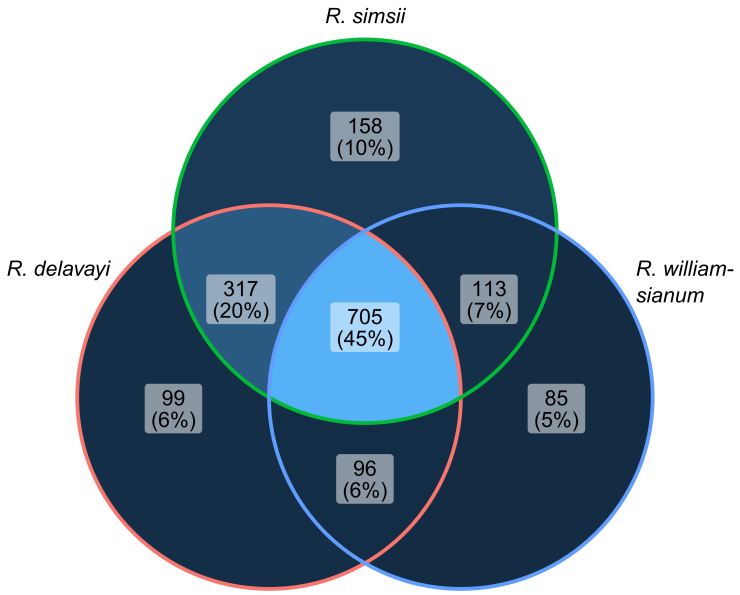

Figure S2 Venn diagram showing the number of genes initially identified by MarkerMiner for each of the three *Rhododendron* transcriptomes.

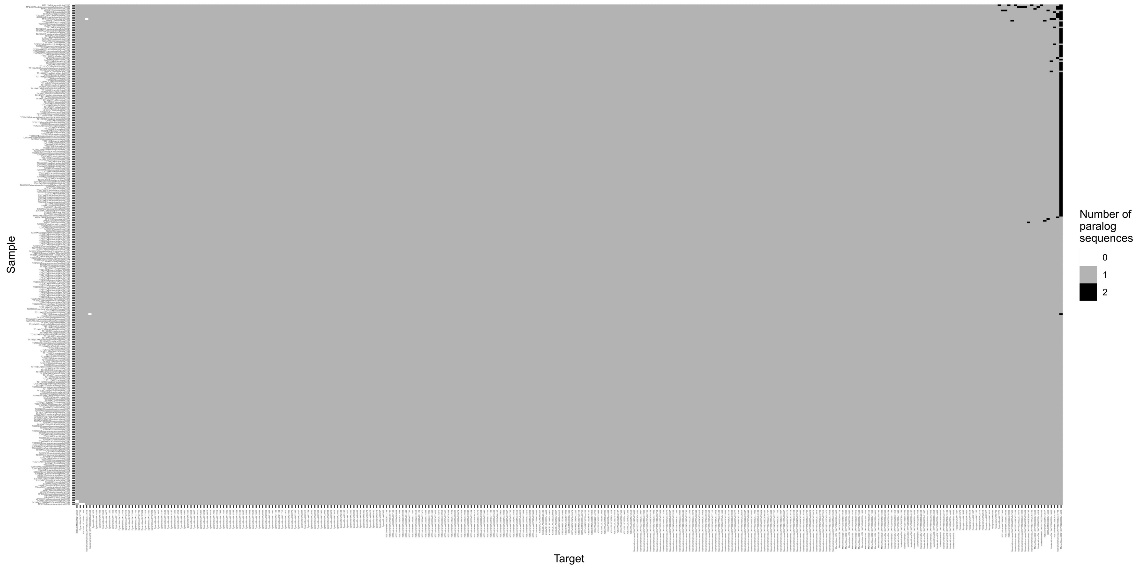

Figure S3 Heatmap showing the number of paralogs (i.e., number of gene copies) identified by HybPiper's length-based method, in which a targets is flagged for a given sample if its second-longest assembled contig is more than 70% the length of its longest assembled contig. Targets and samples are arranged by mean number of copies.

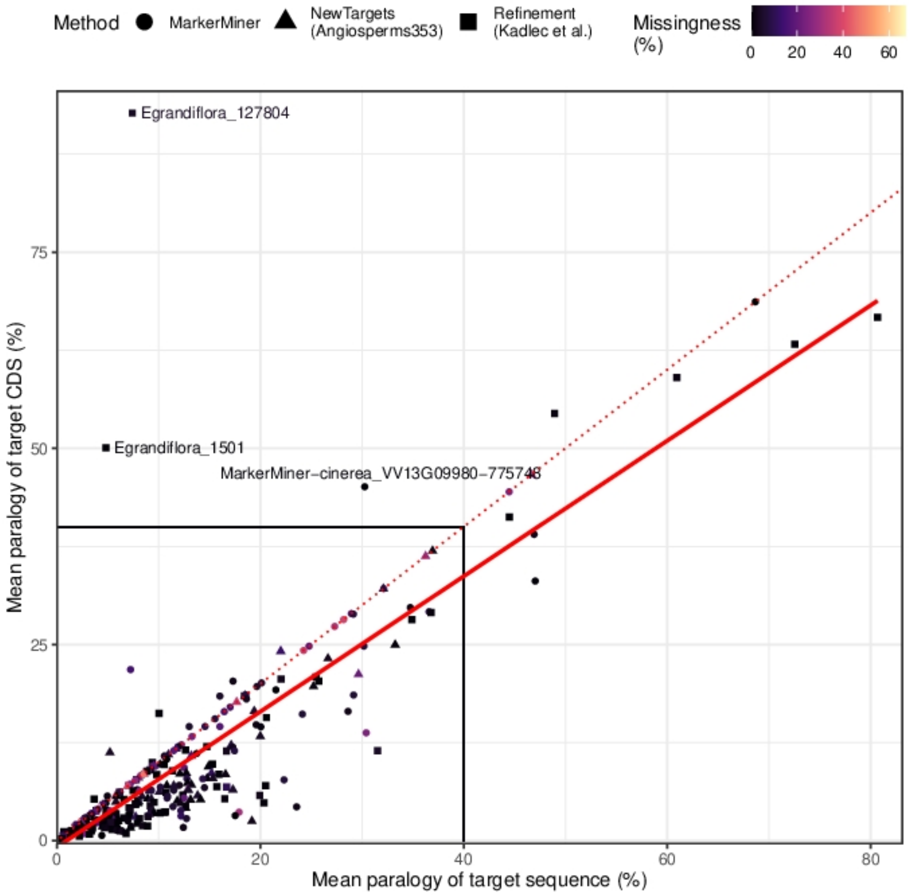

Figure S4 Paralogy (P) estimated using the actual target sequences *versus* using their CDS versions. The solid line shows the linear regression line while the dashed line shows the 1:1 line. Points colours indicate missingness (M).

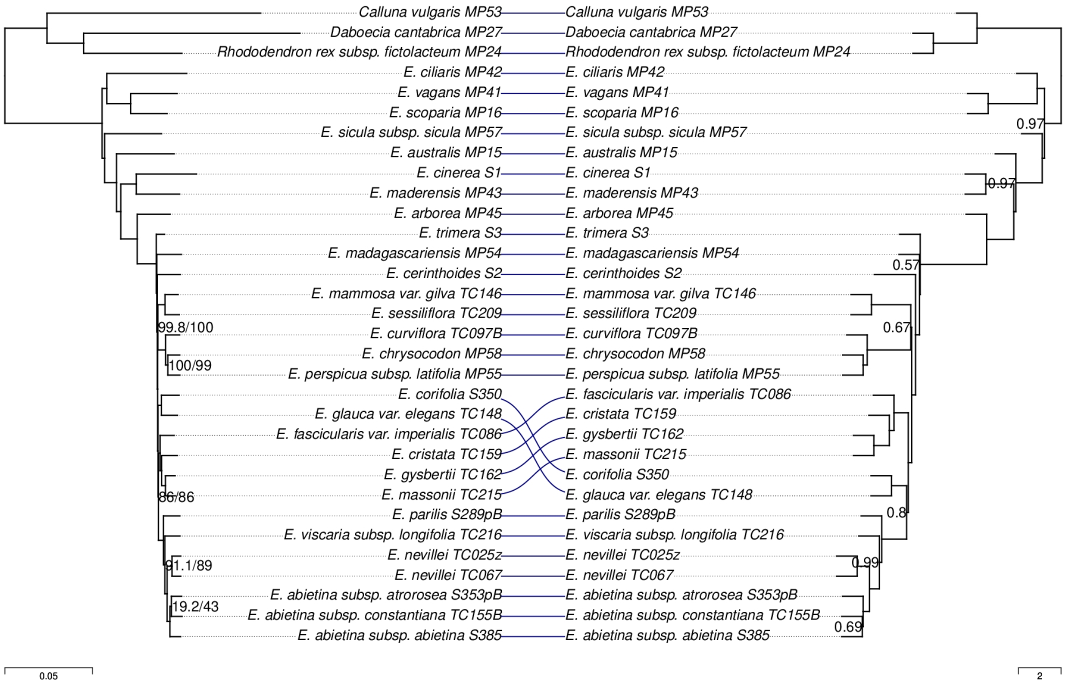

Figure S5 Tanglegram comparing the phylogenies inferred by concatenation (IQ-TREE; *Left*) and by ASTRAL (*Right*) using the full Erica303 target superset. For the concatenation tree, branch lengths are in substitutions per site and node labels are SH-alrt/UFBoot percentages. For the ASTRAL tree, branch lengths represent coalescent units (except for terminal branches which are are arbitrarily set to 1 as they are not estimated by ASTRAL) and node labels show posterior probability support. Nodes with full support are unlabelled. The trees are fully bifurcating and are rooted along the branch between the *Erica* and non-*Erica* samples arbitrarily for display purposes.

2: Supporting Tables

Table S1 Results of the fixed effects models of supercontig length as a function of target source showing that longer supercontigs were recovered by *Erica* genome-derived targets identified using NewTargets and MarkerMiner, whereas longer supercontigs were recovered by *Rhododendron* CDS-derived targets identified using the Refinement method. R^2 indicates the fit of the full model, while Within R^2 indicates the fit when fixed effects are ignored. Numbers in brackets are standard errors.

	MarkerMiner	NewTargets	Refinement
Source = *Rhododendron* CDS: intercept	-1,162.2 bp*** (20.6)	-1,647.2 bp*** (20.7)	1,075.0 bp*** (18.7)
Observations	32,155	23,010	28,910
R^2	0.264	0.176	0.099
Within R^2	0.037	0.077	0.035
Fixed effects			
Sample	✓	✓	✓
Transcript length × Sample	✓	✓	✓

Signif. codes: *** = 0.01, ** = 0.05, * = 0.10

Table S2 Voucher information. Unless otherwise noted, collections were made by the author. Specimens have been deposited at NBG.

Voucher No.	Sample No.	Organism	Latitude	Longitude	iNaturalist*	Collector	Note
SM227	S379B	*E. abietina abietina*	-33.955299	18.424203	25265963	-	-
SM228	S380	*E. abietina abietina*	-33.955805	18.424214	25265904	-	-
SM231	S382	*E. abietina abietina*	-33.955778	18.427173	25265913	-	-
SM269	S384	*E. abietina abietina*	-33.989500	18.413416	28425132	-	-
SM270	S385	*E. abietina abietina*	-33.989421	18.413170	28425133	-	-
SM271	S386	*E. abietina abietina*	-33.988890	18.412935	28425137	-	-
SM272	S387	*E. abietina abietina*	-33.988911	18.411541	28425139	-	-
SM466	S353pB	*E. abietina atrorosea*	-34.090397	18.421659	62543295	-	-
SM475	TC082	*E. abietina atrorosea*	-34.056584	18.372305	63159621	-	-
SM479	TC083	*E. abietina atrorosea* x *E. a. constantiana*	-34.058591	18.374384	63162993	-	-
SM403	TC124	*E. abietina atrorosea* x *E. viscaria viscaria*	-34.101185	18.394171	39853366	-	-
SM415	TC057	*E. abietina constantiana*	-33.999237	18.400214	40627237	-	-
SM416	TC072z	*E. abietina constantiana*	-33.998168	18.400628	40647963	-	-
SM451	TC155B	*E. abietina constantiana*	-34.022580	18.401466	57652605	-	-
SM453	TC063	*E. abietina constantiana*	-34.022344	18.404076	57653130	-	-
SM480	TC065	*E. abietina constantiana*	-34.058591	18.374384	63160250	-	-
SM371	TC043	*E. abietina diabolis*	-33.952053	18.446026	37291642	-	-
SM372	TC123	*E. abietina diabolis*	-33.951908	18.446146	37291782	-	-
SM373	TC053	*E. abietina diabolis*	-33.951878	18.445099	37452995	-	-
SM374	TC028	*E. abietina diabolis*	-33.953216	18.439782	37482859	-	-
SM375	TC184	*E. abietina diabolis*	-33.953356	18.439866	37482860	-	-
SM376	TC044	*E. abietina diabolis*	-33.954363	18.438808	37482861	-	-
SM377	TC116	*E. abietina diabolis*	-33.954329	18.438171	37482863	-	-
SM378	TC045	*E. abietina diabolis*	-33.954038	18.437654	37482864	-	-
SM497	TC052	*E. amphigena*	-34.283811	19.111704	63750814	-	-
SM568	TC211	*E. anguliger*	-34.050745	19.629348	139097180	-	-
EO12619	MP45	*E. arborea*	-	-	-	Ojeda, F (Oliver, EGH)	Sierra del Aljibe, ESP
SM173	TC192	*E. articularis*	-33.995062	18.412987	141363041	Merry, C	-
MP1383	MP15	*E. australis*	-	-	-	Pirie, MD	-
SM214	S277	*E. axilliflora*	-34.698898	19.609428	24777607	-	-
SM436	TC061	*E. axilliflora*	-34.609039	19.560601	54915649	-	-
SM437	S361	*E. axilliflora*	-34.609088	19.560573	54916103	-	-
SM481	TC066	*E. baccans*	-34.058623	18.374230	63160607	-	-
EO12873	MP10	*E. banksii banksii*	-34.2275	19.155139	-	Pirie, MD	-
SM554	TC169pC	*E. brachialis*	-34.352590	18.488490	70803379	-	-
SM611	TC256	*E. bruniifolia*	-34.677857	19.747620	139109850	-	-
SM525	TC101	*E. caffra*	-34.084271	19.056075	68001687	-	-
SM498	TC174z	*E. calycina*	-33.936163	19.162276	64503411	-	-
SM556	TC171	*E. capensis*	-34.258076	18.386052	70803528	-	-
SM137	TC129	*E. cf. borbonifolia*	-34.060626	19.849437	139409785	-	-
SM561	TC204	*E. cf. ericoides*	-34.313326	19.413970	138235130	-	-
SM560	TC203	*E. cf. exleeana*	-34.313943	19.412749	138234900	-	-
SM545	TC160	*E. cf. imbricata*	-34.291926	18.829246	69253541	-	-
SM509	TC180	*E. cf. imbricata*	-33.353301	19.626286	65012535	-	-
SM569	TC212	*E. cf. maritima*	-34.038539	19.623474	139097181	-	-
SM538	TC150	*E. cf. pellucida*	-33.697468	19.114552	69252915	-	-
SM532	TC108	*E. cf. racemosa*	-34.015286	19.109067	68009422	-	-
SM605	TC250	*E. cf. russakiana*	-34.805438	20.036618	139109840	-	-
EO12845	MP58	*E. chrysocodon*	-33.955433	19.174194	-	Oliver, EGH	
MP1377	MP42	*E. ciliaris*	-	-	-	Fagundez, J (Pirie, MD)	Matas de Faja, PRT
SM440	TC081	*E. coccinea coccinea*	-34.639347	19.572571	54967439	-	-
SM570	TC213	*E. coccinea coccinea*	-34.151710	18.926250	139097184	-	-
SM576	TC218pB	*E. coccinea uniflora*	-34.524564	19.449894	139098686	-	-
SM577	TC219pB	*E. coccinea uniflora*	-34.552617	19.416942	139098689	-	-
SM578	TC220pB	*E. coccinea uniflora*	-34.552617	19.416942	139098690	-	-
SM604	TC249	*E. coccinea uniflora*	-34.803242	20.049640	139109837	-	-
SM461	S350	*E. corifolia*	-34.086724	18.423703	58059263	-	-
EO12832	MP4	*E. coventryi*	-	-	-	Oliver, EGH	Fernkloof NR, RSA
SM544	TC159	*E. cristata*	-34.292132	18.829087	69253527	-	-
SM176	TC136z	*E. cruenta*	-33.901626	19.275208	139870023	-	-
SM306	TC141	*E. cruenta*	-34.226385	18.993429	30927319	-	-
SM464	TC097B	*E. curviflora*	-34.093000	18.422442	60396831	-	-
SM603	TC248	*E. curvirostris*	-34.670754	20.042404	139109835	-	-
SM340	TC197pC	*E. curvistyla*	-32.150804	19.027138	32097120	-	-
SM550	TC165pB	*E. cygnea*	-34.286207	18.836168	69253660	-	-
SM551	TC166pB	*E. cygnea*	-34.286174	18.836189	69253697	-	-
SM193	TC088pTC088B	*E. desmantha*	-34.010113	19.005026	21742142	-	-

Continued on next page

Table S2 – continued from previous page

Voucher No.	Sample No.	Organism	Latitude	Longitude	iNaturalist*	Collector	Note
CM19	MP8	*E. diosmifolia*	-33.969111	18.409444	-	Merry, C	-
SM565	TC208	*E. discolor*	-34.317892	19.405846	138235926	-	-
SM392	TC049	*E. doliiformis*	-33.641167	19.132226	37642759	-	-
SM393	TC033	*E. doliiformis*	-33.641478	19.132155	37642862	-	-
SM540	TC152	*E. doliiformis*	-33.689805	19.095086	69252997	-	-
SM541	TC153	*E. doliiformis*	-33.689791	19.095094	69253114	-	-
SM537	TC149	*E. altevivens*	-33.693812	19.148721	68763878	-	-
SM131	TC185pC	*E. embothriifolia longiflora*	-34.064841	19.842018	21954768	-	-
SM496	TC173	*E. eriocephala*	-34.278909	19.118210	63750746	-	-
SM553	TC168	*E. fascicularis*	-34.288848	18.833429	69253740	-	-
SM141	TC086	*E. fascicularis imperialis*	-34.097513	19.849418	139869374	-	-
SM196	TC193pC	*E. fastigiata* (Jonkershoek)	-34.006142	19.008541	21742153	-	-
SM241	TC023pB	*E. filamentosa*	-34.068215	20.482596	26246409	-	-
SM242	TC024	*E. filamentosa*	-34.067938	20.482795	26246416	-	-
SM369	TC095	*E. filiformis*	-34.241425	18.981499	35655599	-	-
SM488	TC119z	*E. flacca*	-32.148443	19.060606	63581885	-	-
SM536	TC148	*E. glauca elegans*	-33.695129	19.148883	69252884	-	-
SM197	TC194pC	*E. glutinosa*	-34.003577	19.011870	21820356	-	-
SM132	TC283	*E. goatcheriana petrensis*	-34.061413	19.844987	21958746	-	-
SM397	TC035	*E. grandiflora grandiflora*	-33.615834	19.099722	38458259	-	-
SM505	TC001	*E. grandiflora grandiflora*	-33.737935	19.077051	65004983	-	-
SM510	TC004z	*E. grandiflora grandiflora*	-33.383728	19.289508	65012667	-	-
SM511	TC036	*E. grandiflora grandiflora*	-33.880461	19.162320	65013572	-	-
SM323	TC115	*E. grandiflora perfoliosa*	-33.992405	18.982022	30184643	-	-
SM171	TC087pTC087B	*E. gysbertii*	-34.365374	18.830066	139419340	-	-
SM547	TC162	*E. gysbertii*	-34.291211	18.831392	69253586	-	-
SM353	TC026	*E. hibbertia*	-33.968671	19.167279	34270237	-	-
SM354	TC143	*E. hibbertia*	-33.968162	19.169098	34270277	-	-
SM363	TC041	*E. hibbertia*	-33.890484	19.333280	35371936	-	-
SM502	TC084	*E. hibbertia*	-33.969402	19.167532	64506172	-	-
SM503	TC068	*E. hibbertia*	-33.968590	19.167484	64506330	-	-
SM167	TC135	*E. imbricata*	-34.356252	18.838414	139419335	-	-
SM303	TC140	*E. imbricata*	-34.112073	18.461566	30366250	-	-
SM409	TC071	*E. imbricata*	-32.721242	18.574251	40598990	-	-
SM490	TC183	*E. imbricata*	-34.293235	19.117652	63582359	-	-
SM533	TC300	*E. imbricata*	-34.015888	19.108541	68009503	-	-
SM558	TC201	*E. imbricata*	-34.313976	19.412756	72014000	-	-
SM527	TC103	*E. intervallaris*	-34.014616	19.108630	68001883	-	-
SM579	TC221pB	*E. irregularis*	-34.524689	19.450186	139098687	-	-
SM580	TC222	*E. irregularis*	-34.524689	19.450167	139098688	-	-
SM507	TC178	*E. junonia minor*	-33.369133	19.657260	65012138	-	-
SM557	TC172	*E. laeta*	-34.272490	18.452146	70865822	-	-
SM368a	TC287	*E. latiflora*	-34.241455	18.981607	35601894	-	-
SM368b	TC288	*E. latiflora*	-34.241455	18.981607	35601894	-	-
SM368c	TC042	*E. latiflora*	-34.241455	18.981607	35601894	-	-
SM342	TC199	*E. limosa*	-34.062023	18.388241	32097556	-	-
SM559	TC202	*E. longiaristata*	-34.313976	19.412756	138234782	-	-
EO12658	MP54	*E. madagascariensis*	-22.162556	46.895194	-	Oliver, EGH	Andringitra N.P., MDG
MP1378	MP43	*E. maderensis*	-	-	-	Fagundez, J (Pirie, MD)	Pico do Areeiro, PRT
SM574	TC217	*E. magnisylvae*	-34.540227	19.429491	139098683	-	-
SM534	TC146	*E. mammosa gilva*	-34.174599	18.387182	69050877	-	-
SM572	TC215	*E. massonii*	-34.151419	18.926541	139097187	-	-
SM535	TC147	*E. melastoma*	-33.694708	19.144099	69051000	-	-
SM341	TC198	*E. mollis*	-34.062117	18.388337	32097555	-	-
SM542	TC157	*E. monadelphia*	-34.296682	18.827416	69252836	-	-
SM530	TC106	*E. multumbellifera*	-34.011308	19.109605	68009210	-	-
EO12747	TC085	*E. nematophylla*	-33.999408	21.283511	-	Oliver, EGH	-
SM348	TC025z	*E. nevillei*	-34.078571	18.371302	34114117	-	-
SM486	TC067	*E. nevillei*	-34.050899	18.366696	63581343	-	-
SM524	TC100	*E. nevillei*	-34.077968	18.371745	66243717	-	-
SM350	TC200	*E. nivea*	-34.078457	18.371172	34176331	-	-
SM160	TC132z	*E. obliqua*	-34.316911	19.008306	139414847	-	-
SM567	TC210	*E. pannosa*	-34.050952	19.627951	138236178	-	-
SM382	TC046	*E. parilis*	-32.957989	19.056368	37581928	-	-
SM395	S289pB	*E. parilis*	-33.879889	19.324957	37642932	-	-
SM506	TC002z	*E. parilis*	-33.353227	19.626078	65005076	-	-
SM501	TC176	*E. penicilliformis*	-33.957409	19.174084	63874419	-	-
SM155	TC187	*E. perspicua*	-34.313096	19.008777	139869377	-	-
EO12844	MP55	*E. perspicua latifolia*	-	-	-	Oliver, EGH	Hermanus area, RSA
SM419	TC073pTC293	*E. petrusiana*	-34.206847	18.841577	40650868	-	-
SM420	TC074pTC294	*E. petrusiana*	-34.206629	18.840715	43550523	-	-
SM421	TC295	*E. petrusiana*	-34.206628	18.840673	43550983	-	-

Continued on next page

Table S2 – continued from previous page

Voucher No.	Sample No.	Organism	Latitude	Longitude	iNaturalist*	Collector	Note
SM422	TC076pTC296	*E. petrusiana*	-34.206937	18.840749	43551418	-	-
SM398	TC096	*E. phillipsii*	-33.636825	19.150734	38267892	-	-
SM400	TC051	*E. phillipsii*	-33.636755	19.149796	38459653	-	-
SM407	TC069	*E. phillipsii*	-32.710507	18.559438	40280913	-	-
SM408	TC070	*E. phillipsii*	-32.725311	18.575945	40593733	-	-
SM161	TC188pC	*E. pillansii*	-34.319722	19.002768	21958778	-	-
SM152	TC285	*E. pinea*	-34.330780	19.015105	139414845	-	-
SM181	S290pB	*E. pinea*	-33.899825	19.268644	21742428	-	-
SM391	TC032	*E. pinea*	-33.627247	19.138071	37615603	-	-
SM394	TC117	*E. pinea*	-33.628470	19.141591	37641574	-	-
SM499	TC009	*E. pinea*	-33.936236	19.162540	64506045	-	-
SM489	TC182	*E. placentiflora*	-34.293235	19.117652	63582250	-	-
SM495	TC099	*E. placentiflora*	-34.278360	19.115821	63711996	-	-
SM331	TC196pC	*E. plukenetii lineata*	-34.657805	19.564903	30757955	-	-
SM531	TC107	*E. plukenetii penicillata*	-34.014398	19.109166	68009345	-	-
SM308	TC195	*E. plumigera*	-34.226597	18.993102	30927320	-	-
SM539	TC151pD	*E. praecox*	-33.690638	19.101909	69252930	-	-
SM508	TC179z	*E. pseudocalycina*	-33.374998	19.665178	65012324	-	-
SM469	S329	*E. pyxidiflora*	-34.179231	18.374584	62543511	-	-
SM333	TC040	*E. quadrisulcata*	-34.238763	18.463073	32097429	-	-
SM387	TC031	*E. quadrisulcata*	-34.213013	18.451676	37615109	-	-
SM388	TC054	*E. quadrisulcata*	-34.213810	18.451488	37615208	-	-
SM389	TC291	*E. quadrisulcata*	-34.214035	18.451405	37615247	-	-
SM390	TC292	*E. quadrisulcata*	-34.214149	18.451327	37615561	-	-
SM218	TC089	*E. regia casta*	-34.705132	19.703732	24777665	-	-
SM612	TC257	*E. regia casta*	-34.705122	19.703773	139109851	-	-
SM441	S364	*E. regia mariae*	-34.423345	20.411664	55131216	-	-
SM608	TC253	*E. regia mariae*	-34.639227	19.925561	139109844	-	-
SM609	TC254	*E. regia mariae*	-34.639229	19.925748	139109847	-	-
SM220	TC022	*E. regia regia*	-34.632403	19.719696	24777684	-	-
SM610	TC255	*E. regia regia*	-34.673109	19.751302	139109849	-	-
SM615	TC260	*E. regia regia*	-34.632246	19.720293	139109856	-	-
SM165	TC134	*E. retorta*	-34.331658	19.009356	139419334	-	-
SM168	TC189	*E. rhopalantha*	-34.361010	18.838370	139419338	-	-
201410903	MP16	*E. scoparia*	-	-	-	Pirie,MD	-
SM504	TC177	*E. cf. imbricata*	-33.969650	19.168905	64026836	-	-
SM617	TC262	*E. cf. placentiflora*	-34.632282	19.720372	139109857	-	-
SM520	TC017	*E. cf. involvens*	-34.365488	18.830055	65705579	-	-
SM521	TC018	*E. cf. involvens*	-34.365588	18.829596	65705827	-	-
SM516	TC015	*E. cf. placentiflora*	-34.523786	19.491117	139508880	-	-
SM500	TC175z	*E. serrata*	-33.957592	19.177049	64028147	-	-
SM571	TC214z	*E. serrata*	-34.151659	18.926727	139097186	-	-
SM438	TC062B	*E. sessiliflora*	-34.631078	19.578888	54916713	-	-
SM566	TC209	*E. sessiliflora*	-34.317884	19.405938	138236031	-	-
Amsn	MP57	*E. sicula sicula*	38.112398	12.665409	-	Pirie, MD	-
SM383	TC029	*E. situshiemalis*	-32.959160	19.070036	37581993	-	-
SM384	TC047	*E. situshiemalis*	-32.959156	19.070643	37614602	-	-
SM385	TC030	*E. situshiemalis*	-32.963753	19.054453	37614694	-	-
SM386	TC048	*E. situshiemalis*	-32.963753	19.054417	37614795	-	-
HLA188	MP51	*E. spiculifolia*	43.368316	22.602508	-	Andersen, HL	-
SM519	TC016pTC297R	*E. stokoei*	-34.364735	18.831755	65705309	-	-
SM485	S377	*E. strigosa*	-34.057282	18.379094	63580879	-	-
SM491	TC005	*E. suffulta*	-34.292374	19.118079	63583296	-	-
SM494	TC008	*E. suffulta*	-34.277872	19.116293	63584397	-	-
SM178	TC137	*E. taxifolia*	-33.899378	19.267509	139417716	-	-
SM170	TC190z	*E. tenella*	-34.364621	18.835220	139419339	-	-
SM156	TC131	*E. tenuifolia*	-34.319827	19.001432	139869375	-	-
2004.0948	MP29	*E. terminalis*	-	-	-	Pirie, MD	Ex. Hort.
SM546	TC161	*E. thomae pink*	-34.291822	18.829595	69253557	-	-
SM425	TC058	*E. thomae tenax*	-34.330193	19.028444	53738251	-	-
SM523	TC299	*E. thomae thomae*	-34.364796	18.831655	65706231	-	-
SM555	TC170	*E. tristis*	-34.352425	18.488271	70803436	-	-
KB_108/01	TC282	*E. turgida*	-	-	-	Lansdowne, A	Ex. Hort.
KB_286/70	TC281	*E. turgida*	-	-	-	Lansdowne, A	Ex. Hort.
SM552	TC167	*E. urceolata*	-34.286912	18.836904	69253722	-	-
MP1376	MP41	*E. vagans*	-	-	-	Fagundez, J (Pirie, MD)	Uzal Capelada, ESP
SM179	TC138	*E. ventricosa*	-33.902820	19.268900	139419341	-	-
SM182	TC139B	*E. ventricosa*	-33.902820	19.268900	139419342	-	-
SM487	TC098pTC098B	*E. verecunda*	-32.148278	19.060391	63581509	-	-
KB_273/12	TC273	*E. verticillata* Adonis	-	-	-	Lansdowne, A	Ex. Hort.
SM583	TC225	*E. verticillata* Adonis	-	-	-	-	Rondevlei, Cape Town
SM592	TC234	*E. verticillata* Adonis	-	-	-	-	Rondevlei, Cape Town

Continued on next page

Voucher No.	Sample No.	Organism	Latitude	Longitude	iNaturalist*	Collector	Note
SM595	TC237	*E. verticillata* Adonis	-	-	-	-	Rondevlei, Cape Town
KB_109/01	TC266	*E. verticillata* Belvedere	-	-	-	Lansdowne, A	Ex. Hort.
SM584	TC226	*E. verticillata* Belvedere	-	-	-	-	Rondevlei, Cape Town
KB_549/06	TC269	*E. verticillata* Cherise	-	-	-	Lansdowne, A	Ex. Hort.
KB_14/12	TC272	*E. verticillata* Dresden	-	-	-	Lansdowne, A	Ex. Hort.
KB_657/06	TC270	*E. verticillata* Harry Wood	-	-	-	Lansdowne, A	Ex. Hort.
SM581	TC223	*E. verticillata* Pretoria	-	-	-	-	Rondevlei, Cape Town
KB_12/12	TC271	*E. verticillata* Rot	-	-	-	Lansdowne, A	Ex. Hort.
KB_543/06	TC267	*E. verticillata* Tresco	-	-	-	Lansdowne, A	Ex. Hort.
KB_548/06	TC268	*E. verticillata* Violet Gray	-	-	-	Lansdowne, A	Ex. Hort.
KB_AL-A	TC274	*E. verticillata* F1	-	-	-	Lansdowne, A	Self-germinated in cult.
KB_AL-B	TC275	*E. verticillata* F1	-	-	-	Lansdowne, A	Self-germinated in cult.
KB_AL-C	TC276	*E. verticillata* F1	-	-	-	Lansdowne, A	Self-germinated in cult.
KB_AL-D	TC277	*E. verticillata* F1	-	-	-	Lansdowne, A	Self-germinated in cult.
KB_AL-E	TC278	*E. verticillata* F1	-	-	-	Lansdowne, A	Self-germinated in cult.
KB_AL-F	TC279	*E. verticillata* F1	-	-	-	Lansdowne, A	Self-germinated in cult.
KB_AL-G	TC280	*E. verticillata* F1	-	-	-	Lansdowne, A	Self-germinated in cult.
SM582	TC224	*E. verticillata* F1	-	-	-	-	Rondevlei, Cape Town
SM585	TC227	*E. verticillata* F1	-	-	-	-	Rondevlei, Cape Town
SM586	TC228	*E. verticillata* F1	-	-	-	-	Rondevlei, Cape Town
SM587	TC229	*E. verticillata* F1	-	-	-	-	Rondevlei, Cape Town
SM588	TC230	*E. verticillata* F1	-	-	-	-	Rondevlei, Cape Town
SM589	TC231	*E. verticillata* F1	-	-	-	-	Rondevlei, Cape Town
SM590	TC232	*E. verticillata* F1	-	-	-	-	Rondevlei, Cape Town
SM593	TC235	*E. verticillata* F1	-	-	-	-	Rondevlei, Cape Town
SM594	TC236	*E. verticillata* F1	-	-	-	-	Rondevlei, Cape Town
SM596	TC238	*E. verticillata* F1	-	-	-	-	Rondevlei, Cape Town
SM597	TC239	*E. verticillata* F1	-	-	-	-	Rondevlei, Cape Town
SM598	TC240	*E. verticillata* F1	-	-	-	-	Rondevlei, Cape Town
SM599	TC241	*E. verticillata* F1	-	-	-	-	Rondevlei, Cape Town
SM600	TC242	*E. verticillata* F1	-	-	-	-	Rondevlei, Cape Town
SM601	TC243	*E. verticillata* F1	-	-	-	-	Rondevlei, Cape Town
SM602	TC244	*E. verticillata* F1	-	-	-	-	Rondevlei, Cape Town
SM219	TC090pTC090B	*E. vestita*	-34.654854	19.694728	24777679	-	-
SM252	TC091	*E. vestita*	-33.952555	20.706150	26246442	-	-
SM253	TC092B	*E. vestita*	-33.950263	20.701460	26246444	-	-
SM512	TC125pTC013	*E. vestita*	-34.534607	19.503297	139508878	-	-
SM515	TC014	*E. vestita*	-34.546746	19.447222	139508879	-	-
SM606	TC251	*E. vestita*	-34.801965	20.037946	139109843	-	-
SM607	TC252	*E. vestita*	-34.801839	20.037688	139508082	-	-
SM613	TC258	*E. vestita*	-34.650245	19.701244	139109852	-	-
SM109	TC110z	*E. viscaria cf. pendula*	-34.167457	19.136064	21592760	-	-
SM427	TC078	*E. viscaria cf. pustulata*	-34.401311	19.282172	53772719	-	-
SM428	TC060	*E. viscaria cf. pustulata*	-34.400735	19.283052	54724558	-	-
SM429	TC265	*E. viscaria cf. pustulata*	-34.396519	19.292284	54724874	-	-
SM431	TC080	*E. viscaria gallorum*	-34.434176	19.575653	54726962	-	-
SM432	S356	*E. viscaria gallorum*	-34.434025	19.575638	54727221	-	-
SM433	S357	*E. viscaria gallorum*	-34.433953	19.575611	54914917	-	-
SM528	TC145	*E. viscaria gallorum*	-34.014667	19.108790	68001972	-	-
SM221	S304pB	*E. viscaria longifolia*	-34.547511	19.634692	24777685	-	-
SM322	TC114	*E. viscaria longifolia*	-34.008756	19.005956	30185492	-	-
SM367	TC027	*E. viscaria longifolia*	-34.242307	18.986215	35619906	-	-
SM396	TC034	*E. viscaria longifolia*	-33.892868	19.342263	37643139	-	-
SM418	TC263	*E. viscaria longifolia*	-34.210136	18.846308	40648767	-	-
SM423	TC264	*E. viscaria longifolia*	-34.195473	18.876924	43552181	-	-
SM430	TC079	*E. viscaria longifolia*	-34.399793	19.277399	54726552	-	-
SM434	S358	*E. viscaria longifolia*	-34.533163	19.529632	54915123	-	-
SM435	S359pB	*E. viscaria longifolia*	-34.533183	19.529095	54915252	-	-
SM526	TC144	*E. viscaria longifolia*	-34.083606	19.056065	68001735	-	-
SM562	TC205	*E. viscaria longifolia*	-34.313078	19.415174	138235357	-	-
SM563	TC206	*E. viscaria longifolia*	-34.313010	19.415478	138235421	-	-
SM573	TC216	*E. viscaria longifolia*	-34.149797	18.927455	139097191	-	-
SM616	TC261	*E. viscaria longifolia*	-34.531837	19.622385	140661283	-	-
SM111	TC021	*E. viscaria macrosepala*	-34.218808	19.185306	21593175	-	-
SM112	TC111	*E. viscaria macrosepala*	-34.218510	19.185201	21593173	-	-
SM150	TC037	*E. viscaria macrosepala*	-34.330859	19.017926	21595097	-	-
SM162	TC113	*E. viscaria macrosepala*	-34.321200	18.995330	139417717	-	-
SM217	TC038	*E. viscaria macrosepala*	-34.699619	19.611001	24777646	-	-
SM424	TC154A	*E. viscaria macrosepala*	-34.329505	19.027246	53736552	-	-
SM426	TC059	*E. viscaria macrosepala*	-34.218796	19.185254	53738686	-	-
SM439	S363	*E. viscaria macrosepala*	-34.639183	19.572540	54916867	-	-
SM614	TC259	*E. viscaria macrosepala*	-34.649675	19.700340	139109854	-	-
SM309	TC039	*E. viscaria pendula*	-34.226613	18.992817	30927323	-	-

Continued on next page

Table S2 – continued from previous page

Voucher No.	Sample No.	Organism	Latitude	Longitude	iNaturalist*	Collector	Note
SM310	TC127	*E. viscaria pendula*	-34.226667	18.992743	30927328	-	-
SM492	TC006	*E. viscaria pendula*	-34.293777	19.117615	63582891	-	-
SM493	TC007	*E. viscaria pendula*	-34.287821	19.107834	63584232	-	-
SM460	TC064	*E. viscaria viscaria*	-34.086622	18.424147	58058820	-	-
SM462	S351pB	*E. viscaria viscaria*	-34.086711	18.423764	58059688	-	-
SM463	S352pB	*E. viscaria viscaria*	-34.086430	18.423797	60396570	-	-
SM468	S328	*E. viscaria viscaria*	-34.181278	18.370693	62543403	-	-
SM564	TC207	*E. xeranthemifolia*	-34.312461	19.417026	138235606	-	-
W-2013.0655-01	MP53	*Calluna vulgaris*	60.498116	4.915009	-	Moe, B	-
W-1999.0498	MP27	*Daboecia cantabrica*	-	-	-	Pirie, MD	León, ESP
W-1996.0626	MP24	*Rhododendron rex fictolacteum*	-	-	-	Pirie, MD	Beima Shan, CHN

*iNaturalist observations can be viewed at `inaturalist.org/observations/<iNaturalistID>`.
